# Association between cardiometabolic disease multimorbidity and all-cause mortality in 2 million women and men registered in UK general practices

**DOI:** 10.1186/s12916-021-02126-x

**Published:** 2021-10-28

**Authors:** Dexter Canoy, Jenny Tran, Mariagrazia Zottoli, Rema Ramakrishnan, Abdelaali Hassaine, Shishir Rao, Yikuan Li, Gholamreza Salimi-Khorshidi, Robyn Norton, Kazem Rahimi

**Affiliations:** 1 Nuffield Department of Women’s and Reproductive Health, University of Oxford, Hayes House, 75 George St., OX1 2BQ Oxford, UK; 2 NIHR Oxford Biomedical Centre, Oxford University Hospitals NHS Foundation Trust, Oxford, UK; 3 Department of Statistics, University of Oxford, Oxford, UK; 4 The George Institute for Global Health, University of New South Wales, Sydney, Australia

**Keywords:** Myocardial infarction, Stroke, Diabetes, Multimorbidity, Mortality, Electronic health records

## Abstract

**Background:**

Myocardial infarction (MI), stroke and diabetes share underlying risk factors and commonalities in clinical management. We examined if their combined impact on mortality is proportional, amplified or less than the expected risk separately of each disease and whether the excess risk is explained by their associated comorbidities.

**Methods:**

Using large-scale electronic health records, we identified 2,007,731 eligible patients (51% women) and registered with general practices in the UK and extracted clinical information including diagnosis of myocardial infarction (MI), stroke, diabetes and 53 other long-term conditions before 2005 (study baseline). We used Cox regression to determine the risk of all-cause mortality with age as the underlying time variable and tested for excess risk due to interaction between cardiometabolic conditions.

**Results:**

At baseline, the mean age was 51 years, and 7% (*N* = 145,910) have had a cardiometabolic condition. After a 7-year mean follow-up, 146,994 died. The sex-adjusted hazard ratios (*HR*) (95% confidence interval [*CI*]) of all-cause mortality by baseline disease status, compared to those without cardiometabolic disease, were MI = 1.51 (1.49–1.52), diabetes = 1.52 (1.51–1.53), stroke = 1.84 (1.82–1.86), MI and diabetes = 2.14 (2.11–2.17), MI and stroke = 2.35 (2.30–2.39), diabetes and stroke = 2.53 (2.50–2.57) and all three = 3.22 (3.15–3.30). Adjusting for other concurrent comorbidities attenuated these estimates, including the risk associated with having all three conditions (*HR* = 1.81 [*95% CI* 1.74–1.89]). Excess risks due to interaction between cardiometabolic conditions, particularly when all three conditions were present, were not significantly greater than expected from the individual disease effects.

**Conclusion:**

Myocardial infarction, stroke and diabetes were associated with excess mortality, without evidence of any amplification of risk in people with all three diseases. The presence of other comorbidities substantially contributed to the excess mortality risks associated with cardiometabolic disease multimorbidity.

**Supplementary Information:**

The online version contains supplementary material available at 10.1186/s12916-021-02126-x.

## Background

The prevalence of multimorbidity, defined as having two or more long-term conditions [1], has been increasing, a global trend partly driven by increasingly ageing population and improved survival from major causes of mortality [2–4]. However, the impact of multimorbidity on mortality remains unclear. The combined impact of different conditions on mortality risk could simply reflect the sum of the total effects of each condition, or it could be less than the sum of individuals effects of each condition, particularly when coexisting conditions are concordant, that is, when co-occurring diseases are likely to share aetiology, predisposing factors or clinical management. Alternatively, the presence of a condition might exacerbate the impact of another such that the combined effect of the different conditions is substantially higher (or lower) than would normally be expected from the separate effects of each disease.

Myocardial infarction, diabetes mellitus and stroke are cardiometabolic diseases that are prevalent and among the leading causes of mortality globally [5, 6]. Others have reported increased mortality risk in those with more than one of these cardiometabolic conditions (Additional file 1: Table S1) [7–20]. However, these studies have been based on small numbers of deaths, did not compare risk with those without cardiometabolic disease and did not explore the impact of other coexisting chronic conditions. Considering that these diseases are largely concordant, we hypothesised that, whilst the mortality risks of patients with two or all three of these conditions will be high, the combined risk will not be more than the excess deaths expected from each individual cardiometabolic condition, which could have implications on managing the conditions of these people with multimorbidity.

In addition, multimorbid individuals have been thought to be at increased risk of mortality particularly those with higher numbers of coexisting conditions [12, 21–25]. Since people with myocardial infarction, diabetes and stroke tend to also have other long-term conditions [4, 19, 26, 27], the presence of these additional comorbidities could potentially influence mortality risk in people with cardiometabolic disease multimorbidity. We therefore examined the separate and combined associations of having myocardial infarction, diabetes and stroke with all-cause mortality and assessed the impact of having other comorbidities on these associations.

## Methods

### Data source

We conducted this study using linked electronic health records from the Clinical Practice Research Datalink (CPRD) [28] which collects de-identified patient data from a network of general practices across the UK. The CPRD provided data for this research covering the period from its inception in 1985 up to 2015. At the time of conducting this study, the CPRD database contained data from 674 general practices, which covered around 7% of the UK population and broadly represented the population by age, sex and ethnicity [29]. This database is linked to other national administrative databases including hospitalisations (Hospital Episode Statistics [30]), death registration (Office of National Statistics [31]) and the Index of Multiple Deprivation [32], which makes the CPRD database a comprehensive resource for prospective analysis of people registered with general practices in the UK. The validity and reliability of recorded diagnoses, including cardiovascular disease and diabetes, have been reported previously [33, 34]. The CPRD Independent Scientific Advisory Committee has given scientific approval for this study (Protocol number 16_049R), and no separate informed consent was required to access data for this research.

### Study population and period of follow-up

We identified patients in the database meeting the following criteria: (1) registered with the general practice for ≥12 months, (2) aged ≥16 years at registration, (3) registered with the practice considered providing ‘up-to-standard’ data to CPRD, (4) individual data marked by CPRD to be of ‘acceptable’ quality for research purposes, (5) registered with their practice that provided consent for data linkage with national databases for hospitalisations and death registry and (6) had a ‘valid’ record with their practice by 1 January 2005. As conditions tend to be overreported a few months shortly after registration with the general practice [35], we allowed for a lag time of at least 12 months, and only considered patients’ recorded data after the first 12 months of their current registration with the practice as ‘valid’ for prospective follow-up from baseline. We extracted data on demographics and clinical history for each patient up until 31 Dec 2004 and ascertained vital status for the whole cohort during follow-up as provided by CPRD. Thus, we created a patient cohort of 2,007,731 women and men who entered into the study on 1 Jan 2005, with baseline clinical history extracted prior to this date, and followed up until death, exit from the practice or censored at the end of follow-up (by 31 Dec 2014).

### Definition of cardiometabolic conditions, comorbidities and other variables

We used a previously reported list of long-term conditions considered to be clinically significant and prevalent in the UK [4], which were selected from the (1) Quality and Outcomes Framework, an incentive scheme for general practitioners in the UK [36]; (2) Charlson comorbidity index which is a widely used index of comorbidity which was originally designed for predicting in-hospital mortality [37]; and (3) list of multiple chronic conditions of the US Department of Health and Human Services Initiative on Multiple Chronic Conditions [38]. We used a list of diagnostic codes from hospital records, based on the International Classification of Diseases 10th revision (ICD-10), and primary care record, based on the Read coding scheme [39], to identify relevant diagnoses as described previously [4]. Myocardial infarction, diabetes and stroke were the three cardiometabolic conditions that we were primarily interested to investigate as these common conditions are major causes of mortality globally. The diagnostic codes for these conditions are listed in Table S2 (Additional file 1). We classified the study cohort according to their cardiometabolic disease status at baseline in mutually exclusive groups. The remaining 53 chronic conditions were considered as additional comorbidity based on the first recording of a diagnostic code in a patient’s record appearing before baseline (Additional file 1: Table S3 and mapping of Read codes to chronic conditions) [4]. We extracted information including demographic data, clinical information (hypertension, dyslipidaemia and obesity), smoking status and deprivation level based on the Index of Multiple Deprivation, which provides an area-based indicator of relative deprivation ranked from least to most deprived fifth at the national level.

### Statistical analysis

We described the characteristics of the cohort and their distributions according to disease status at baseline. We used Cox regression to estimate the hazard ratio of mortality for each cardiometabolic condition relative to those without any of these conditions at baseline. We verified the proportional hazard assumption by plotting the Kaplan-Meier survival curve and performing the Schoenfeld residual analysis. We used age as the underlying time variable and showed models that adjusted for sex, smoking and deprivation level, then additionally for the other 53 comorbidities at baseline. As the number of conditions or groups of related conditions is predictive of mortality [12, 21–25], we also conducted analyses that operationalised baseline comorbidities according to the number of coexisting conditions (0, 1, 2, 3, 4 or ≥5 additional comorbidities) and broadly related categories of these comorbidities. As a sensitivity analysis, we also show results by taking into account additional comorbidities occurring after baseline and show results separately for men and women and by attained age (<75 and ≥75 years). We described the ethnicity, hypertension, dyslipidaemia and body mass index to provide contextual information of our patient cohort, but we did not use these variables in any further analyses as the cohort was predominantly of white ethnicity, and considered the other variables as mediating risk factors.

We estimated absolute risks by calculating the adjusted mortality rate for each disease (see Additional file 1: Supplementary Method). We then calculated excess deaths by taking the difference in the adjusted mortality rate between a comparator (e.g. patients with stroke) and the reference group (e.g. patients without any of the cardiometabolic diseases). We investigated the combined effects of myocardial infarction, diabetes and stroke on mortality by assessing the hazard ratios derived from Cox models for deviations from multiplicativity (the ratio of the risk associated with the combined effect of two or more factors over the product of the risks of the individual factors) and additivity (the relative excess risk due to interaction) [40–42]. Using these methods, an interaction between two or three cardiometabolic diseases would be demonstrated by showing death rates that are higher (or lower) than expected from the death rates associated with each condition alone.

We conducted our analyses using the R statistical software (version 3.6.1) [43]. To account for missing data, we implemented the *mice* package in R to perform 15 imputations (fraction of missing information < 8 × 10^−6^) and ran our analyses on 15 imputed datasets. We presented hazard ratios with their 95% group-specific confidence interval (*CI*) [44] to allow comparison of risks between two groups even if neither of the two was the reference category when calculating the hazard ratio. We considered two-sided *P* values < 0.05 as statistically significant.

## Results

In this cohort of 2,007,731 (51% women), the mean (standard deviation [*SD*]) baseline age was 51.4 (*SD* = 17.5) years, and 7.3% (*N* = 145,910) have had a diagnosis of at least one of the cardiometabolic diseases of interest (Table 1). The proportions of those with a single condition were 3.6%, 1.7% and 1.3% for diabetes, myocardial infarction and stroke, respectively; for those with two conditions, the proportions were 0.4% for diabetes and myocardial infarction, 0.2% for diabetes and stroke and 0.1% for myocardial infarction and stroke; for those with all three conditions, the proportion was 0.05%. Compared to those without cardiometabolic disease at baseline, those with the disease were older, had higher deprivation level and were more likely to be ever smokers. Except for those with only stroke at baseline, the proportions of women were lower than those of men in all other cardiometabolic disease status at baseline. Table 1 also shows that patients with cardiometabolic disease were more likely to have a higher number of additional comorbidities. For example, the proportions of those with five or more comorbidities in patients with all cardiometabolic conditions were 25%; in contrast, among those without any cardiometabolic disease at baseline, the proportion was only 2%.

**Table 1:** Characteristics of patients, according to cardiometabolic disease status at baseline

	No cardiometabolic disease at baseline	With cardiometabolic disease at baseline							All patients
		Myocardial infarction	Diabetes	Stroke	Myocardial infarction and diabetes	Myocardial infarction and stroke	Stroke and diabetes	Myocardial infarction, stroke and diabetes	
*No. of patients (% women)*	*1,861,821 (52)*	*33,581 (30)*	*71,399 (47)*	*25,136 (53)*	*7206 (30)*	*2901 (36)*	*4753 (46)*	*934 (33)*	*2,007,731 (51)*
Baseline characteristics									
Mean (*SD*) age (years)	50.0 (17.0)	71.4 (11.7)	64.5 (14.3)	73.7 (13.5)	71.3 (10.5)	76.8 (10.2)	73.8 (10.6)	75.1 (9.1)	51.4 (17.5)
% White ethnicity (*n*)	96 (579,570)	98 (15,845)	94 (27,511)	98 (11,538)	95 (3004)	99 (1407)	95 (2003)	97 (417)	96 (641,295)
[*N*]	[*601,722*]	[*16,150*]	[*29,260*]	[*11,743*]	[*3154*]	[*1426*]	[*2100*]	[*432*]	[*665,987*]
% Hypertension (*n*)	34 (598,162)	61 (20,232)	65 (46,582)	69 (17,094)	68 (4,864)	68 (1959)	76 (3599)	72 (669)	37 (693,161)
[*N*]	[*1,736,780*]	[*33,443*]	[*71,180*]	[*24,931*]	[*7199*]	[2*887*]	[*4736*]	[*929*]	[*1,882,085*]
% Dyslipidaemia (*n*)	50 (438,350)	18 (4802)	18 (10,623)	25 (4289)	13 (772)	18 (373)	16 (588)	14 (101)	46 (459,898)
[*N*]	[*876,937*]	[*27,191*]	[*59,942*]	[16,842]	[*6040*]	[*2124*]	[*3647*]	[*724*]	[*993,447*]
% Most deprived fifth (*n*)	14 (258,571)	17 (5607)	18 (12,535)	17 (4322)	19 (1400)	18 (530)	20 (971)	22 (201)	14 (284,137)
[*N*]	[*1,857,578*]	[*33,500*]	[*71,387*]	[*25,087*]	[*7196*]	[*2897*]	[*4746*]	[*934*]	[*2,003,225*]
% BMI ≥30 kg/m^2^ (*n*)	16 (194,941)	20 (5451)	42 (25,661)	18 (3153)	39 (2510)	17 (393)	36 (1417)	35 (284)	17 (233,809)
[*N*]	[*1,235,911*]	[*27,421*]	[*61,687*]	[*17,934*]	[*6414*]	[*2244*]	[*3911*]	[*816*]	[*1,356,338*]
% Ever smokers (*n*)	43 (585,436)	63 (18,309)	48 (29,466)	50 (9,863)	61 (3957)	59 (1428)	51 (2083)	58 (486)	44 (651,028)
[*N*]	[*1,351,139*]	[*29,268*]	[*61,711*]	[*19,904*]	[*6475*]	[*2437*]	[*4055*]	[*833*]	[*1,475,822*]
No. with other comorbidities, % (*n*)									
None	52 (964,906)	21 (7068)	30 (21,397)	17 (4283)	16 (1139)	9 (253)	14 (664)	7 (64)	50 (999,774)
One	26 (491,284)	24 (8233)	28 (19,673)	24 (5983)	21 (1527)	16 (479)	22 (1038)	14 (132)	26 (528,349)
Two	12 (227,813)	20 (6817)	19 (13,319)	21 (5344)	21 (1493)	18 (536)	22 (1046)	20 (187)	13 (256,.555)
Three	6 (102,475)	14 (4784)	11 (8014)	16 (4101)	16 (1176)	19 (562)	17 (787)	18 (170)	6 (122,069)
Four	2 (44,217)	9 (3092)	6 (4580)	10 (2553)	10 (757)	15 (430)	11 (505)	16 (146)	3 (56,280)
Five or more	2 (31,126)	11 (3587)	6 (4416)	11 (2872)	16 (1114)	22 (641)	15 (713)	25 (235)	2 (44,704)
Follow-up information									
Mean (SD) follow-up (year)	7.1 (3.2)	6.3 (3.3)	6.9 (3.2)	5.4 (3.5)	5.9 (3.3)	4.7 (3.4)	5.1 (3.4)	4.5 (3.2)	7.0 (3.3)
Person-years (1000s)	13,178	211	494	137	42	14	24	4	14,105
No. of deaths	85,117	15,233	22,582	14,184	3961	2082	3121	714	146,994
Mean (*SD*) age at death (years)	78.7 (13.6)	82.1 (9.4)	79.3 (11.0)	83.9 (9.4)	79.7 (9.2)	83.0 (8.5)	81.0 (9.0)	80.3 (8.1)	79.3 (12.9)
Crude rate per 10,000 per year	143.3	721.5	456.9	1037.9	927.2	1540.3	1279.9	1719.0	177.8

*BMI* body mass index. Data in [] are numbers with missing information for the variable of interest

Over an average of 7 years of follow-up, 146,994 died (crude mortality rate = 104.2 deaths per 10,000 per year), with the mean age at death = 79.3 (*SD* = 12.9) years.

**Fig. 1:**
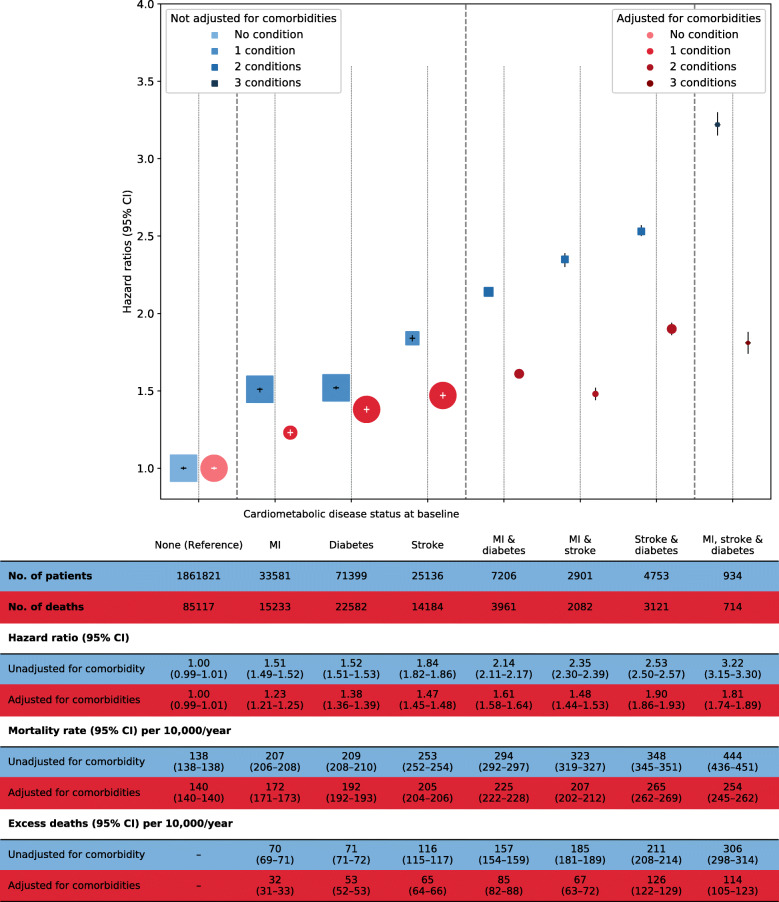
Risk of death according to cardiometabolic disease status at baseline. Risk estimates based on Cox regression with age as the underlying time variable and adjusted for sex with and without further adjustment for 53 additional comorbidities. The area of the square or circle is inversely proportional to the variance of the log risk. In the figure, error bars include the estimate (horizontal bar) and CI (vertical bar). *CI* confidence interval

Figure 1 (and Table S4) shows the risk of death associated with baseline disease status. Adjusting for sex, smoking and deprivation, each single condition was associated with increased risk of mortality, with the highest risk seen in patients with stroke only (hazard ratio = 1.84 [*95% CI* 1.82 to 1.86]) when compared to those without any of the cardiometabolic condition at baseline. In absolute terms, there were 116 (*95% CI* 115 to 117) excess deaths per 10,000 per year in the former when compared to the latter group. When two conditions were present, there were doubling of risks relative to those without any of the cardiometabolic diseases. The risk was highest when all three diseases were present (hazard ratio = 3.22 [*95% CI* 3.15 to 3.30]), which was associated with excess deaths of 306 (*95% CI* 298 to 314) per 10,000 per year. After additionally adjusting for other comorbidities at baseline, the risks associated with myocardial infarction, stroke and diabetes, when present individually or in combination, were attenuated. In particular, when all three conditions were present, the hazard ratio was attenuated to 1.81 (*95% CI* 1.74 to 1.89) and the absolute excess risk to 114 (95% 105 to 123) per 10,000 per year when compared to those without any of the condition. The risk estimates remained similar when we additionally adjusted for comorbidities occurring after baseline (Table S4). The results remained consistent in women and men, in attained ages <75 and ≥75 years (Additional file 1: Tables S5 and S6). The impact of adjusting for comorbidities operationalised by the number of coexisting conditions as well as by broadly-related categories of chronic conditions are shown in Table 2.

**Table 2:** Risk of death according to cardiometabolic disease status at baseline, adjusting for comorbidity using alternative indicators for this variable

	No cardiometabolic disease at baseline	With cardiometabolic disease at baseline						
		Myocardial infarction	Diabetes	Stroke	Myocardial infarction and diabetes	Myocardial infarction and stroke	Stroke and diabetes	Myocardial infarction, stroke and diabetes
No. of persons	1,861,821 (52)	33,581 (30)	71,399 (47)	25,136 (53)	7206 (30)	2901 (36)	4753 (46)	934 (33)
No. of deaths	85,117	15,233	22,582	14,184	3961	2082	3121	714
Hazard ratio (*95% CI*)								
Adjusted for sex, smoking, deprivation and comorbidities at baseline	1.00 (0.99 to 1.01)	1.21 (1.19 to 1.23)	1.34 (1.32 to 1.35)	1.47 (1.44 to 1.49)	1.59 (1.55 to 1.63)	1.46 (1.41 to 1.52)	1.86 (1.81 to 1.90)	1.72 (1.63 to 1.81)
Adjusted for sex, smoking, deprivation and number of comorbidities^a^ at baseline	1.00 (0.99 to 1.01)	1.18 (1.16 to 1.19)	1.32 (1.31 to 1.33)	1.14 (1.13 to 1.16)	1.47 (1.43 to 1.50)	1.24 (1.19 to 1.28)	1.37 (1.34 to 1.41)	1.43 (1.36 to 1.50)
Adjusted for sex, smoking, deprivation and categories of comorbidities^b^ at baseline	1.00 (0.99 to 1.01)	1.27 (1.26 to 1.29)	1.40 (1.39 to 1.41)	1.46 (1.44 to 1.48)	1.73 (1.70 to 1.76)	1.58 (1.54 to 1.63)	1.91 (1.88 to 1.95)	2.13 (2.05 to 2.20)

*CI* confidence interval; hazard ratios based on Cox regression using age as the underlying time variable; ^a^ 0, 1, 2, 3, 4 and ≥5 comorbidities; ^b^ comorbidities categorised as cardiometabolic (but not myocardial infarction, diabetes or stroke), mental health, respiratory, musculoskeletal, neurological, cancers and others

**Fig. 2:**
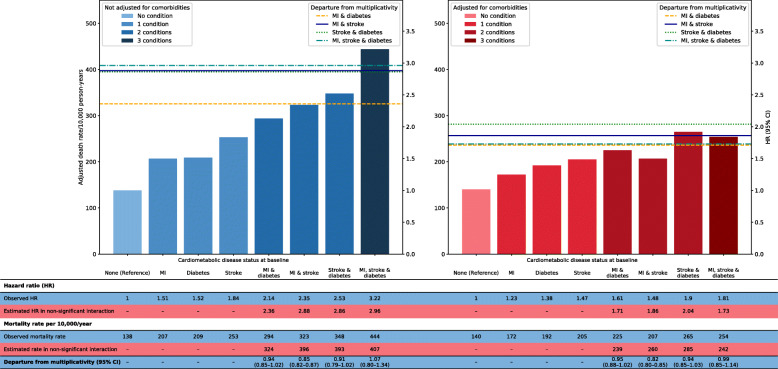
Assessing interaction of two or more cardiometabolic conditions on mortality risk and departure from a multiplicative scale. All risk estimates based on Cox regression with age as underlying time variable and adjusted for sex with and without further adjustment for 53 additional comorbidities. Each coloured line represents expected risk estimates when no significant interaction (that is, no departure from multiplicativity) exists between two or more cardiometabolic diseases. *HR* hazard ratio, *CI* confidence interval, MI myocardial infarction

We examined for any synergistic or antagonistic effects on mortality when two or more of the cardiometabolic conditions were present (Fig. 2). There was no evidence that the presence of two or more conditions resulted in more deaths than expected from mortality risk attributable to each condition. For example, if an interaction between diabetes and stroke results in a combined risk higher than expected from the excess deaths associated with each condition separately, the excess risk should be higher than 393 deaths per 10,000 per year; yet the excess deaths in this patient group was 348 (*95% CI* 345 to 351) deaths per 10,000 per year (Fig. 2—left panel). We observed similar patterns when we additionally adjusted for baseline comorbidities (Fig. 2—right panel). There is some evidence of an interaction on an additive scale in the presence of both diabetes and stroke (Additional file 1: Fig. S1—left panel), which was attenuated, but not eliminated, after taking into account other baseline comorbidities (Additional file 1: Fig. S1—right panel). Interestingly, there was departure from multiplicativity associated with the presence of both myocardial infarction and stroke (Fig. 2), whether or not other comorbidities were adjusted for. The mortality risk was lower than expected from the risks associated separately with either disease, suggesting an ‘antagonistic’ pattern of interaction. We did not see any significant relative excess risk when all three conditions were present; if anything, the excess risk was lower than expected from the sum of the risks of each condition after adjusting for the additional comorbidities (Additional file 1: Fig. S1—right panel).

## Discussion

In this cohort of over two million women and men, people with a history of myocardial infarction, diabetes or stroke had an increased risk of mortality. These increased risks were, in part, explained by the presence of additional comorbidities. Those with more than one of the cardiometabolic diseases had a higher risk than those with one or no cardiometabolic condition. Whilst the co-occurrence of two or more of these conditions resulted in substantially high excess deaths, the risks were not generally higher than would have been expected from the impact of the individual disease.

Comparison of risks with previous studies is difficult due to varying cardiometabolic conditions being considered (Additional file 1: Table S1) although our study broadly supports earlier findings in showing that mortality risk is high in patients with a history of cardiometabolic disease, particularly when these diseases co-occur. However, findings showing the combined impact of myocardial infarction, diabetes and stroke are limited as previous studies have been based on small numbers of deaths [12, 17], lacked data for comparison with people without any of the cardiometabolic conditions [9] or did not take into account other coexisting comorbidities [9, 12, 15, 17]. Unlike most of these studies, we specifically explored the impact of co-occurring cardiometabolic conditions on mortality risk and examined the importance of other coexisting long-term conditions in contributing to the risk of death.

A previous report showed a multiplicative effect, suggesting an amplification of mortality risk associated with cardiometabolic disease multimorbidity [15]. By pooling data from several prospective cohort studies, mortality associated with a history of heart disease, diabetes and stroke—separately for those with one, two or all of these conditions. The investigators suggest that participants with two or more conditions have substantially increased mortality risk than would have been expected from the mortality rates associated with each disease. Their seemingly discrepant findings from those in our study could be due to differences in the study population. In their investigation, the study population included cohort participants who were younger and more likely to have healthier background risk than our study population identified from general practices. Some of the cohorts in the collaborative study collected baseline data in the 1960s and 1970s, whereas our study population was based on registration with general practices in more recent decades. As mortality risk associated with cardiometabolic disease, when compared to those without the condition, has been decreasing in recent years [13, 14, 26], our estimated mortality risks are likely to reflect those of patients in relatively more contemporary settings whose clinical management differed from those in earlier generations. Our study also differed in that we were able to take into account other comorbidities that patients with myocardial infarction, diabetes and stroke also had, and we have demonstrated that in such patients these additional long-term conditions have a substantial impact on their mortality risk.

Our observation that the excess deaths were not amplified in those with multiple cardiometabolic conditions is consistent with our hypothesis that, when concordant conditions coexist, the overall risk is unlikely to be greater than the sum of the individual effects of each condition. Clinical factors, such as elevated blood pressure and dyslipidaemia, are common in these patients, and treatment of these risk factors typically forms part of their clinical care. Our findings could reflect the concordance in the pathophysiology, clinical features and management of vascular and metabolic disease and are consistent with research indicating that people with multimorbidity are more likely to receive evidence-based treatment than expected [45–47]. These results highlight the importance of managing and treating these risk factors to modify mortality outcomes in patients with coexisting vascular and metabolic diseases.

Furthermore, our study also showed the impact of other additional comorbidities on the mortality of patients with cardiometabolic disease. These additional comorbidities could be indicative of the disease burden these patients have, either in terms of their number or type of comorbidity (e.g. cancer-related condition) [21, 24, 25]. It is also plausible that patients with these additional comorbidities have conditions that are ‘discordant’ (e.g. different aetiology), for which they may have received suboptimal care [48]. Indeed, the presence of multiple conditions could potentially affect the quality of care patients with cardiovascular disease or diabetes receive [49–51]. Yet, there is also evidence that when these discordant conditions are identified, such as during clinical assessment to identify these conditions when this evaluation forms part of routine care for multimorbid patients or opportunistically during clinical encounters, these patients could actually receive better care [12, 45–47]. Whilst we found some evidence that of an interaction between myocardial infarction and stroke when assessed on an additive scale, the excess risk was largely attenuated when other comorbidities were taken into account. Our findings therefore highlight the importance of other coexisting chronic conditions in influencing mortality risk of people with cardiometabolic disease multimorbidity.

There are a number of considerations when interpreting our results. We used routine practice data, which could be prone to recording errors and biases that include differential recording of diagnoses. We addressed these potential issues with appropriate considerations in designing the study, such as limiting analyses to records flagged to be of research quality standards and imposing restrictions on the minimum duration of registration with the general practice [35]. We neither validated nor adjudicated diagnoses but recorded diagnoses of cardiometabolic disease in the CPRD database have been shown to have high validity [33]. We were unable to distinguish type 1 from type 2 diabetes as the specific type is frequently not recorded in the database. The duration of the cardiometabolic diseases and the timing and sequence of the occurrence of the comorbidities could not be precisely determined as there could be delays between making the diagnoses of multiple chronic conditions and their recording. We also did not have information on other lifestyle factors, such as physical activity level, alcohol consumption and diet, which are not routinely recorded in healthcare databases. Although our study population is predominantly of white ethnicity, patients included in the CPRD database are representative of the UK general population in terms of age, sex and ethnicity [29]. Our findings may not be applicable in acute settings, as our study population would include those who would have had survived at least the first disease event for a duration long enough to be considered in our analysis. Finally, the extent to which our findings are applicable to people with other chronic conditions and discordant comorbidities, or other healthcare settings, requires further investigation. Nevertheless, an important strength of our study is the scale, volume and size of the data, including sufficient duration of follow-up to accrue sufficient numbers of events across the different multimorbidity subgroups.

## Conclusion

In this large population of women and men, myocardial infarction, stroke and diabetes, separately and in combination, were associated with excess mortality which was partly due to associated additional comorbidities. We found no evidence that the co-occurrence of all three cardiometabolic conditions contributed to a higher excess mortality than expected from each of them separately, indicating that mortality risk is not necessarily amplified in cardiometabolic disease multimorbidity but rather modifiable. The excess risk was largely related to the presence of other comorbidities, underscoring the need for a wholistic approach when assessing and evaluating risks in people with multimorbidity.

## Supplementary Information

**Additional file 1: Supplementary method**. Estimating adjusted mortality rates. **Table S1**. Studies comparing mortality risk according to cardiometabolic conditions. **Table S2**. The ICD-10 and Read codes used to define cardiometabolic disease. **Table S3**. List of additional comorbidities. **Table S4**. Risk of death according to cardiometabolic disease status at baseline with and without adjustment for comorbidities. **Table S5**. Risk of death according to cardiometabolic disease status at baseline, stratified by sex. **Table S6**. Risk of death according to cardiometabolic disease status at baseline, stratified by age. **Fig. S1**. Assessing interaction of two or more cardiometabolic conditions on mortality risk on an additive scale (relative excess risk due to interaction). **Mapping of Read codes to chronic conditions**.

## Data Availability

Data underlying this article were provided with permission from the CPRD (www.cprd.com); similar data may be requested directly from them.

## Abbreviations

*CI*: Confidence interval
CPRD: Clinical Practice Research Datalink
ICD-10: International Classification of Diseases 10^th^ Revision
*SD*: Standard deviation
UK: United Kingdom
