# Supplementary material for: Association between cardiometabolic disease multimorbidity and all-cause mortality in 2 million women and men registered in UK general practices
Source: BMC Med. 2021 Oct 28;19:258. doi: 10.1186/s12916-021-02126-x (PMC8555122; doi:10.1186/s12916-021-02126-x)
Supplement: Supplementary file 1 — Additional file 1: Supplementary method. Estimating adjusted mortality rates. Table S1. Studies comparing mortality risk according to cardiometabolic conditions. Table S2. The ICD-10 and Read codes used to define cardiometabolic disease. Table S3. List of additional comorbidities. Table S4. Risk of death according to cardiometabolic disease status at baseline with and without adjustment for comorbidities. Table S5. Risk of death according to cardiometabolic disease status at baseline, stratified by sex. Table S6. Risk of death according to cardiometabolic disease status at baseline, stratified by age. Fig. S1. Assessing interaction of two or more cardiometabolic conditions on mortality risk on an additive scale (relative excess risk due to interaction). Mapping of Read codes to chronic conditions. [file 12916_2021_2126_MOESM1_ESM.docx]

Additional file

**Association between cardiometabolic disease multimorbidity and all-cause mortality in 2 million women and men registered in UK general practices**

Dexter Canoy^1,2,3^, Jenny Tran^1,2^, Mariagrazia Zottoli^1,4^, Rema Ramakrishnan^1,2,4^, Abdelaali Hassaine^1,2^, Shishir Rao^1,2^, Yikuan Li^1,2^, Gholamreza Salimi-Khorshidi^1,2^, Robyn Norton^5^, Kazem Rahimi^1-3^

^1^ Deep Medicine, Oxford Martin School, University of Oxford, Oxford, UK

^2^ Nuffield Department of Women’s and Reproductive Health, University of Oxford, Oxford, UK

^3^ NIHR Oxford Biomedical Centre, Oxford University Hospitals NHS Foundation Trust, Oxford, UK

^4^ Department of Statistics, University of Oxford, UK

^5^ The George Institute for Global Health, University of New South Wales, Sydney, Australia

Contents

**Supplementary method** Estimating adjusted mortality rates (Page 2)

**Table S1** Studies comparing mortality risk according to cardiometabolic conditions (Page 3)

**Table S2** The ICD-10 and Read codes used to define cardiometabolic disease (Page 4)

**Table S3** List of additional comorbidities (Page 5)

**Table S4** Risk of death according to cardiometabolic disease status at baseline with and without adjustment for comorbidities (Page 6)

**Table S5** Risk of death according to cardiometabolic disease status at baseline, stratified by sex (Page 7)

**Table S6** Risk of death according to cardiometabolic disease status at baseline, stratified by age (Page 8)

**Fig. S1** Assessing interaction of two or more cardiometabolic conditions on mortality risk on an additive scale (relative excess risk due to interaction) (Page 9)

**Mapping of Read codes to chronic conditions** (Page 10)

**Supplementary method**

**Estimating adjusted mortality rates**

To calculate adjusted mortality rate for each disease status, we multiplied the adjusted hazards ratios for each of the cardiometabolic disease category (obtained from the Cox regression models) to the mortality rate of the reference category, and divided the product by the weighted average of each cardiometabolic condition-specific hazard ratio by the person-years for each category, as shown in Equation (1):

(1)

$$\frac{{HR}_{i} *\frac{d_{ref}}{N_{ref}}}{\frac{1}{N}\sum\mathrm{HR}_{i}w_{i}}$$

Where:

*HR_i_ = Hazard Ratio for the i_th_ cardiometabolic condition*

$\frac{d_{ref}}{N_{ref}}$ *= Death rate for the reference category (No cardiometabolic disease, where*

*d_ref_ = Number of deaths in the reference category*

*N_ref_ = Person-years at risk in the reference category*

$\frac{1}{N}\sum HRi w_{i}$ *= Weighted average of the stratum category- specific hazard, where:*

*N = Person-years at risk in the population*

*HR_i_ = Hazard ratio for the i_th_ stratum, and*

*w_i_ = Total person-years at risk for patients in the i_th_ stratum*

**Table S1** Studies comparing mortality risk according to cardiometabolic conditions

| Study | Study population (No. of events) | Cardiometabolic conditions compared | Comments |
| --- | --- | --- | --- |
| Ostgren [7] (2002) | 400 patients with type 2 diabetes; 131 died over 5.9 years of follow-up | Diabetes and CVD | No comparison with no cardiometabolic disease; impact of additional comorbidities not investigated |
| Hu [8] (2005) | 51,735 cohort participants; 9201 died over 17.2 years of follow-up | MI and diabetes | Impact of additional comorbidities not investigated |
| Lee [9] (2007) | 741,847 veteran health care users; 52,306 died over 5 years of follow-up | Diabetes, CHD and stroke | No comparison with no cardiometabolic disease; impact of additional comorbidities not investigated; hypertension included in cardiometabolic disease cluster |
| Glynn [10] (2008) | 1609 patients with CVD registered in general practices; 141 died after 5 years of follow-up | Diabetes and CVD | No comparison with no cardiometabolic disease; Comorbidities adjusted for were limited to other vascular conditions |
| Schramm [11] (2008) | 3.2 million Danish residents; 287,471 died over 5 years of follow-up | MI and diabetes | Impact of additional comorbidities not investigated |
| Caughey [12] (2010) | 2087 cohort participants; 1469 died over 14 years of follow-up | Diabetes and CVD (heart attack, TIA and stroke) | Cardiometabolic disease cluster included arthritis; impact of additional comorbidities not investigated |
| Schmidt [13] (2012) | 234,331 patients with first MI hospitalisation (1984 to 2008); one-year standardised mortality rate at 42% 1984-8 falling to 24% in 2004-8 | MI, diabetes and stroke | No comparison with no cardiometabolic disease; Impact of additional comorbidities not investigated (except for stratified analysis by categories based on Charlson comorbidity index) |
| Schmidt [14] (2014) | 219,354 patients with first stroke hospitalisation (1994 to 2011); five-year standardised mortality rate approximately 50% | Stroke, MI and diabetes | No comparison with no cardiometabolic disease; Impact of additional comorbidities not investigated (except for stratified analysis by categories based on Charlson comorbidity index) |
| ERFC [15] (2015) | ERFC cohort: 689,300 participants; 128,843 died over 12.8 years (median) follow-up; UK Biobank cohort: 499,808 participants; 7995 died over 4.8 years (median) follow-up | MI, diabetes and stroke | Impact of additional comorbidities not investigated |
| Rashid [16] (2017) | Systematic review | Stroke and CHD | Compared patients according to Charlson comorbidity index categories (most common comorbidities were prior MI and diabetes); No comparison with no cardiometabolic disease |
| Kang [17] (2017) | 569,831 cohort participants; 50,249 died over 12 years (median) follow-up | MI, diabetes and stroke | Only 19 died in those with all three conditions; Impact of additional comorbidities, except for chronic kidney disease, not investigated |
| Hall [18] (2018) | 693,388 patients with MI; 204,667 died over 5 years of follow-up | MI and diabetes | No comparison with no cardiometabolic disease; Impact of additional comorbidities not investigated |
| Gallacher [19] (2018) | 8751 cohort participants with stroke/TIA; 735 died over 7 years of follow-up | Stroke, diabetes and CHD | No comparison with no cardiometabolic disease; Impact of additional comorbidities not investigated |
| Zhang [20] (2019) | 1.04 million health care registered patients; 22,750 died over 5.2 years (median) follow-up | Diabetes and CVD (CHD and stroke) | Impact of additional comorbidities not investigated |

CVD – cardiovascular disease; MI – myocardial infarction; CHD – coronary heart disease or ischaemic heart disease; TIA – transient ischaemic attack; ERFC – Emerging Risk Factors Collaboration.

**Table S2** The ICD-10 and Read codes used to define cardiometabolic disease

| Disease | Code |
| --- | --- |
|  |  |
| **Myocardial infarction** |  |
| ICD-10 | I20, I20.0, I20.8, I20.9, I21, I21.0, I21.1, I22.2, I21.3, I21.4, I21.9, I22, I22.0, I22.1, I22.8, I22.9, I23.7, I25, I25.0, I25.1, I25.2, I25.3, I25.4, I25.5, I25.6, I25.7, I25.8, I25.9, I21, I21.0, I21.1, I21.2, I21.3, I21.4, I21.9, I22, I22.0, I22.1, I22.2, I22.8, I22.9, I23, I23.0, I23.1, I23.2, I23.3, I23.4, I23.5, I23.6, I23.8, I24.1 |
|  |  |
| Read | 241, 1204, 1677, 1678, 2491, 3704, 4017, 5387, 7783, 8935, 9507, 10562, 12139, 12229, 13566, 13571, 14658, 14897, 14898, 15661, 16408, 17133, 17464, 17689, 17872, 18842, 23579, 23708,, 23892, 24126, 26972, 26975, 28736, 29553, 29643, 29758, 30330, 30421, 32272, 32854, 34803, 35119, 35674, 36423, 37657, 38609, 40399, 40429, 41221, 41835, 45809, 46017, 46112, 46166, 46276, 50372, 52705, 55401, 59032, 59189, 59940, 61670,  62626, 63467, 68357, 68748, 69474, 72562, 96838 |
|  |  |
| **Stroke** | |
| ICD-10 | G46.3, G46.4, G46.5, G46.6, G46.7, I60, I61, I62.0, I62.1, I62.9, I63, I64, I69.0, I69.1, I69.2, I69.3, I69.4, I69.8 |
|  |  |
| Read | 1298, 1469, 3132, 3535, 5051, 5185, 5363, 5871, 6116, 6155, 6228, 6253, 6305, 6960, 7138, 7780, 7912, 8443, 10792, 10962, 11074, 12833, 13564, 17322, 17326, 18604, 18686, 18687, 19201, 19348, 19412, 28314, 28753, 28914, 30045, 30202, 31060, 31218, 31595, 32959, 33499, 33543, 34135, 34245, 34375, 39403, 40053, 40338, 40758, 42248, 43451, 46316, 47607, 47642, 48149, 51465, 51767, 52246, 53745, 53810, 55351, 56007, 56279, 56458, 57183, 57315, 66873, 70536, 89913, 90572, 91627, 92036, 93459, 94482, 95347, 96630 |
| **Diabetes** | |
| ICD-10 | E10, E11, E12, E13, E14, G59.0, G63.2, H28.0, H36.0, M14.2, N08.3, O24.0, O24.1, O24.2, O24.3 |
|  |  |
| Read | 758, 1407, 1549, 10418, 10692, 12455, 12640, 12736, 17262, 17545, 17858, 17859, 18143, 18209, 18219, 18230, 18264, 18278, 18387, 18390, 18425, 18496, 18642, 18683, 18777, 21983, 22871, 22884, 24423, 24458, 24836, 25591, 25627, 26054, 30294, 30323, 32627, 34268, 34450, 35288, 35385, 36633, 37648, 37806, 38161, 39070, 40682, 40837, 41049, 42729, 42762, 42831, 43227, 43921, 44440, 44779, 44982, 45913, 45914, 45919, 46150, 46301, 46850, 46917, 47315, 47321, 47409, 47582, 47649, 47650, 47816, 47954, 48192, 49074, 49146, 49554, 49655, 49869, 49949, 50225, 50527, 50813, 51756, 51957, 53392, 54008, 54899, 55075, 55239, 56268, 57278, 58604, 59253, 59725, 60107, 60208, 60699, 60796, 61071, 61344, 61829, 62107, 62209, 62352, 62613, 62674, 63017, 63690, 64571, 64668, 65267, 65704, 66145, 66872, 66965, 67905, 68105, 68390, 69676, 69993, 70316, 70766, 85991, 91646, 91942, 91943, 93468, 93727, 93878, 95343, 95351, 95992, 96235, 97446, 97474, 97894, 98616, 98723, 99231, 104323, 102201, 100292 |
|  |  |

ICD-10 - World Health Organization's International Classification of Diseases, Tenth Revision.

**Table S3** List of additional comorbidities [4]

| Group | Comorbidity | Included long-term conditions |
| --- | --- | --- |
| Cardiometabolic | Cardiac arrhythmia | Cardiac arrhythmias and dysrhythmias, including atrial fibrillation and flutter, paroxysmal tachycardia, atrioventricular blocks, and other conduction disorders |
|  | Chronic kidney disease | Chronic kidney disease stage 3 or more, or dependence on transplant or dialysis |
|  | Heart failure |  |
|  | Peripheral arterial disease | Includes aortic aneurysm and dissection, embolism and thrombosis, and unspecified peripheral vascular diseases |
| Mental health | Adjustment disorder | Includes pathological reactions to severe stress |
|  | Affective disorder | Unspecified, persistent, or other affective disorders |
|  | Anxiety | Anxiety and phobic disorders |
|  | Bipolar disorder |  |
|  | Depression | Depression and depressive mood disorders |
|  | Eating disorder | Eating disorders, including bulimia and anorexia nervosa |
|  | Psychoses | Psychotic and delusional disorders |
|  | Schizophrenia |  |
|  | Substance abuse | Mental and behavioural disorders due to substances including alcohol, opioids, and cannabinoids |
| Respiratory | Asthma |  |
|  | COPD |  |
|  | Lung cancer |  |
|  | Other respiratory cancer | Respiratory organ cancer excluding lung cancer, e.g., laryngeal cancer, pleural cancer, and tracheal cancer |
| Musculoskeletal | Arthritis | Chronic arthritis, including osteoarthritis, secondary arthritides (e.g., arthropathy in Crohn’s disease and psoriatic arthritis), and other unspecified arthritides and sequelae; excluding acute arthritis (e.g., septic arthritis) and arthritides covered elsewhere: gout, rheumatoid arthritis, and connective-tissue-related arthritis |
|  | Gout |  |
|  | Osteoporosis |  |
|  | Rheumatoid arthritis |  |
| Neurological | Dementia | Includes Alzheimer’s disease, vascular dementia, and unspecified dementia |
|  | Epilepsy |  |
|  | Hemiplegia |  |
|  | Learning disability | Disorders of psychological development, mental retardation, and childhood autism |
| Cancers | Bladder cancer |  |
|  | Breast cancer |  |
|  | Cervical cancer |  |
|  | Colon cancer |  |
|  | Ear, nose, and throat cancer |  |
|  | Leukaemia |  |
|  | Liver cancer |  |
|  | Lymphoma |  |
|  | Metastatic cancer |  |
|  | Oesophageal cancer |  |
|  | Ovarian cancer |  |
|  | Pancreatic cancer |  |
|  | Prostate cancer |  |
|  | Rectal cancer |  |
|  | Renal cancer |  |
|  | Skin cancer |  |
|  | Stomach cancer |  |
|  | Other female reproductive  cancer | Female reproductive organ cancer excluding breast, ovarian, and cervical cancer, e.g., endometrial cancer, uterine cancer, and cancer of the vagina |
|  | Other gastrointestinal | Gastrointestinal cancer excluding oesophageal, stomach/gastric, colon, rectal, pancreatic, and liver cancer, e.g., cancer of the bile ducts, anal cancer, and peritoneal cancer |
|  | Other male reproductive cancer | Male reproductive organ cancer excluding prostate cancer, e.g., testicular cancer |
|  | Other urological cancer | Urological organ cancer excluding renal/kidney and bladder cancer, e.g., cancer of the ureter and cancer of the urethra |
|  | Unspecified cancer | Cancer with unspecified anatomical region or origin |
|  | Other cancer | Cancer not otherwise covered by all other cancers covered in the list of 56 comorbidities, e.g., brain cancer, cancer of the eye, and cancer of the endocrine glands |
| Other diseases | Connective tissue disease | Systemic connective tissue disorders including systemic lupus erythematosus, systemic sclerosis, and polymyositis |
|  | HIV/AIDS |  |
|  | Liver disease | Includes cirrhosis, portal hypertension, and hepatic failure; excludes liver cancer |
|  | Peptic ulcer disease | Includes gastric and duodenal ulcers |

Excluded: Diabetes mellitus (including type I and II, and diabetes-specific sequelae), hypertension (includes hypertensive-specific sequelae), hyperlipidaemia and obesity; COPD – chronic obstructive pulmonary disease; HIV/AID – human immunodeficiency virus/Acquired Immunodeficiency Syndrome.

**Table S4** Risk of death according to cardiometabolic disease status at baseline with and without adjustment for comorbidities.

|  | No cardiometabolic  disease at baseline | With cardiometabolic disease at baseline | | | | | | |
| --- | --- | --- | --- | --- | --- | --- | --- | --- |
|  |  | **Myocardial**  **infarction** | **Diabetes** | **Stroke** | **Myocardial infarction and diabetes** | **Myocardial**  **infarction**  **and stroke** | **Stroke and**  **diabetes** | **Myocardial**  **Infarction, stroke**  **and diabetes** |
| No. of patients | 1,861,821 (52) | 33,581 (30) | 71,399 (47) | 25,136 (53) | 7206 (30) | 2901 (36) | 4753 (46) | 934 (33) |
| No. of deaths | 85,117 | 15,233 | 22,582 | 14,184 | 3961 | 2082 | 3121 | 714 |
| Excess deaths per 10,000 per year (95% CI) | |  |  |  |  |  |  |  |
| Adjusted for sex, smoking  and deprivation level | 0* | 69.6  (68.7 to 70.6) | 71.5  (70.9 to 72.0) | 115.7  (114.9 to 116.5) | 156.9  (154.2 to 159.4) | 185.1  (181.1 to 189.2) | 210.7  (207.9 to 213.5) | 306.1  (298.5 to 313.6) |
| Additionally adjusted for  baseline comorbidities | 0† | 32.2  (31.1 to 33.3) | 52.6  (52.1 to 53.2) | 65.0  (64.0 to 66.0) | 85.4  (82.4 to 88.3) | 67.4  (62.6 to 72.0) | 125.6  (122.3 to 128.8) | 113.8  (105.0 to 122.5) |
| Additionally adjusted for  incident comorbidities | 0‡ | 32.9  (31.8 to 33.9) | 59.2  (58.7 to 59.7) | 65.4  (644 to 66.3) | 86.8  (83.9 to 89.6) | 73.8  (69.2 to 78.4) | 134.1  (130.9 to 137.2) | 137.2  (128.7 to 145.7) |
| Hazard ratio (95% CI) | |  |  |  |  |  |  |  |
| Adjusted for sex, smoking  and deprivation level | 1.00 (0.99 to 1.01) | 1.51 (1.49 to 1.52) | 1.52 (1.51 to 1.53) | 1.84 (1.82 to 1.86) | 2.14 (2.11 to 2.17) | 2.35 (2.30 to 2.39) | 2.53 (2.50 to 2.57) | 3.22 (3.15 to 3.30) |
| Additionally adjusted for  baseline comorbidities | 1.00 (0.99 to 1.01) | 1.23 (1.21 to 1.25) | 1.38 (1.36 to 1.39) | 1.47 (1.45 to 1.48) | 1.61 (1.58 to 1.64) | 1.48 (1.44 to 1.53) | 1.90 (1.86 to 1.93) | 1.81 (1.74 to 1.89) |
| Additionally adjusted for  incident comorbidities | 1.00 (0.99 to 1.01) | 1.24 (1.22 to 1.25) | 1.42 (1.41 to 1.44) | 1.47 (1.45 to 1.49) | 1.62 (1.59 to 1.65) | 1.53 (1.49 to 1.57) | 1.96 (1.93 to 2.00) | 1.98 (1.91 to 2.06) |

CI – confidence interval; Hazard ratios based on Cox regression using age as the underlying time variable; *Mortality rate=137.6 (137.5 to 137.7) per 10,000 per year; †Mortality rate=139.8 (95% CI 139.8 to 139.9) per 10,000 per year; ‡Mortality rate=139.6 ((95% CI 139.5 to 139.6) per 10,000 per year.

**Table S5** Risk of death according to cardiometabolic disease status at baseline, stratified by sex

|  | No cardiometabolic  disease at baseline | With cardiometabolic disease at baseline | | | | | | |
| --- | --- | --- | --- | --- | --- | --- | --- | --- |
|  |  | **Myocardial**  **infarction** | **Diabetes** | **Stroke** | **Myocardial infarction and diabetes** | **Myocardial**  **infarction**  **and stroke** | **Stroke and**  **diabetes** | **Myocardial**  **Infarction, stroke**  **and diabetes** |
| MEN |  |  |  |  |  |  |  |  |
| No. of persons | 899,174 | 23,477 | 37,768 | 11,788 | 5012 | 1857 | 2583 | 624 |
| No. of deaths | 85,117 | 9706 | 11,413 | 6376 | 2586 | 1290 | 1651 | 460 |
| Person-years (1000s) | 6,326,976 | 152,342 | 263,387 | 66,500 | 30,337 | 9066 | 13,688 | 2908 |
| Hazard ratio (95% CI) | |  |  |  |  |  |  |  |
| Adjusted for smoking and deprivation level | 1.00 (0.99 to 1.01) | 1.46 (1.44 to 1.48) | 1.48 (1.46 to 1.49) | 1.85 (1.83 to 1.88) | 2.06 (2.02 to 2.10) | 2.36 (2.30 to 2.41) | 2.47 (2.42 to 2.52) | 3.04 (2.95 to 3.13) |
| Additionally adjusted for baseline comorbidities | 1.00 (0.99 to 1.01) | 1.21 (1.19 to 1.23) | 1.34 (1.32 to 1.35) | 1.47 (1.44 to 1.49) | 1.59 (1.55 to 1.63) | 1.46 (1.41 to 1.52) | 1.86 (1.81 to 1.90) | 1.72 (1.63 to 1.81) |
| WOMEN, N (No. of deaths) |  |  |  |  |  |  |  |  |
| No. of persons | 962,647 | 10,104 | 33,631 | 13,348 | 2194 | 1044 | 2170 | 310 |
| No. of deaths | 103,803 | 5527 | 11,169 | 7808 | 1375 | 792 | 1470 | 254 |
| Person-years (1000s) | 6,851,311 | 58,796 | 230,857 | 70,150 | 12,381 | 4451 | 10,698 | 1246 |
| Hazard ratio (95% CI) | |  |  |  |  |  |  |  |
| Adjusted for smoking and deprivation level | 1.00 (0.99 to 1.01) | 1.58 (1.56 to 1.61) | 1.56 (1.54 to 1.58) | 1.82 (1.80 to 1.84) | 2.26 (2.21 to 2.32) | 2.33 (2.26 to 2.40) | 2.58 (2.53 to 2.63) | 3.61 (3.49 to 3.73) |
| Additionally adjusted for baseline comorbidities | 1.00 (0.99 to 1.01) | 1.27 (1.24 to 1.29) | 1.41 (1.39 to 1.43) | 1.45 (1.43 to 1.48) | 1.62 (1.57 to 1.68) | 1.54 (1.47 to 1.61) | 1.92 (1.87 to 1.97) | 2.08 (1.95 to 2.20) |

CI – confidence interval; Hazard ratios based on Cox regression using age as the underlying time variable.

**Table S6** Risk of death according to cardiometabolic disease status at baseline, stratified by age

| Age group | No cardiometabolic  disease at baseline | With cardiometabolic disease at baseline | | | | | | |
| --- | --- | --- | --- | --- | --- | --- | --- | --- |
|  |  | **Myocardial**  **infarction** | **Diabetes** | **Stroke** | **Myocardial infarction and diabetes** | **Myocardial**  **infarction**  **and stroke** | **Stroke and**  **diabetes** | **Myocardial infarction, stroke**  **and diabetes** |
| LESS THAN 75 years |  |  |  |  |  |  |  |  |
| No. of persons | 1,690,844 | 20,277 | 54,307 | 12,082 | 4554 | 1175 | 2551 | 448 |
| No. of deaths | 63,978 | 3338 | 7054 | 2272 | 1149 | 368 | 805 | 188 |
| Person-years (1000s) | 12,215,081 | 145,370 | 403,347 | 82,328 | 30,274 | 7092 | 15,711 | 2416 |
| Hazard ratio (95% CI) | |  |  |  |  |  |  |  |
| Adjusted for smoking and deprivation level | 1.00 (0.99 to 1.01) | 2.05 (2.02 to 2.08) | 1.99 (1.97 to 2.01) | 2.58 (2.54 to 2.62) | 3.18 (3.13 to 3.24) | 4.03 (3.92 to 4.13) | 4.03 (3.96 to 4.10) | 4.87 (4.73 to 5.01) |
| Additionally adjusted for baseline comorbidities | 1.00 (0.99 to 1.01) | 1.39 (1.35 to 1.42) | 1.62 (1.59 to 1.64) | 1.58 (1.54 to 1.63) | 1.75 (1.69 to 1.81) | 1.48 (1.38 to 1.59) | 2.39 (2.31 to 2.46) | 1.54 (1.40 to 1.69) |
| 75 YEARS AND OLDER |  |  |  |  |  |  |  |  |
| No. of persons | 317,886 | 20,650 | 31,409 | 17,195 | 4386 | 2212 | 3184 | 657 |
| No. of deaths | 124,942 | 11,895 | 15,528 | 11,912 | 2812 | 1714 | 2316 | 526 |
| Person-years | 2,240,227 | 126,109 | 211,220 | 87,320 | 25,969 | 10,088 | 16,187 | 2923 |
| Hazard ratio (95% CI) | |  |  |  |  |  |  |  |
| Adjusted for smoking and deprivation level | 1.00 (0.99 to 1.01) | 1.42 (1.40 to 1.44) | 1.39 (1.37 to 1.40) | 1.73 (1.72 to 1.75) | 1.90 (1.86 to 1.94) | 2.15 (2.10 to 2.19) | 2.21 (2.17 to 2.25) | 2.80 (2.72 to 2.89) |
| Additionally adjusted for baseline comorbidities | 1.00 (0.99 to 1.01) | 1.19 (1.17 to 1.20) | 1.28 (1.27 to 1.30) | 1.45 (1.43 to 1.46) | 1.48 (1.44 to 1.52) | 1.50 (1.45 to 1.55) | 1.74 (1.70 to 1.78) | 1.76 (1.68 to 1.85) |

CI – confidence interval; Hazard ratios based on Cox regression with age as the underlying time variable.

**Fig. S1** Assessing interaction of two or more cardiometabolic conditions on mortality risk on an additive scale (relative excess risk due to interaction [RERI])

All risk estimates based on Cox regression with age as underlying time variable and adjusted for sex. Each coloured line represents expected risk estimates when no significant interaction (ie, no departure from additivity) exist between two or more cardiometabolic diseases. HR – hazard ratio; CI – confidence interval; MI – myocardial infarction.


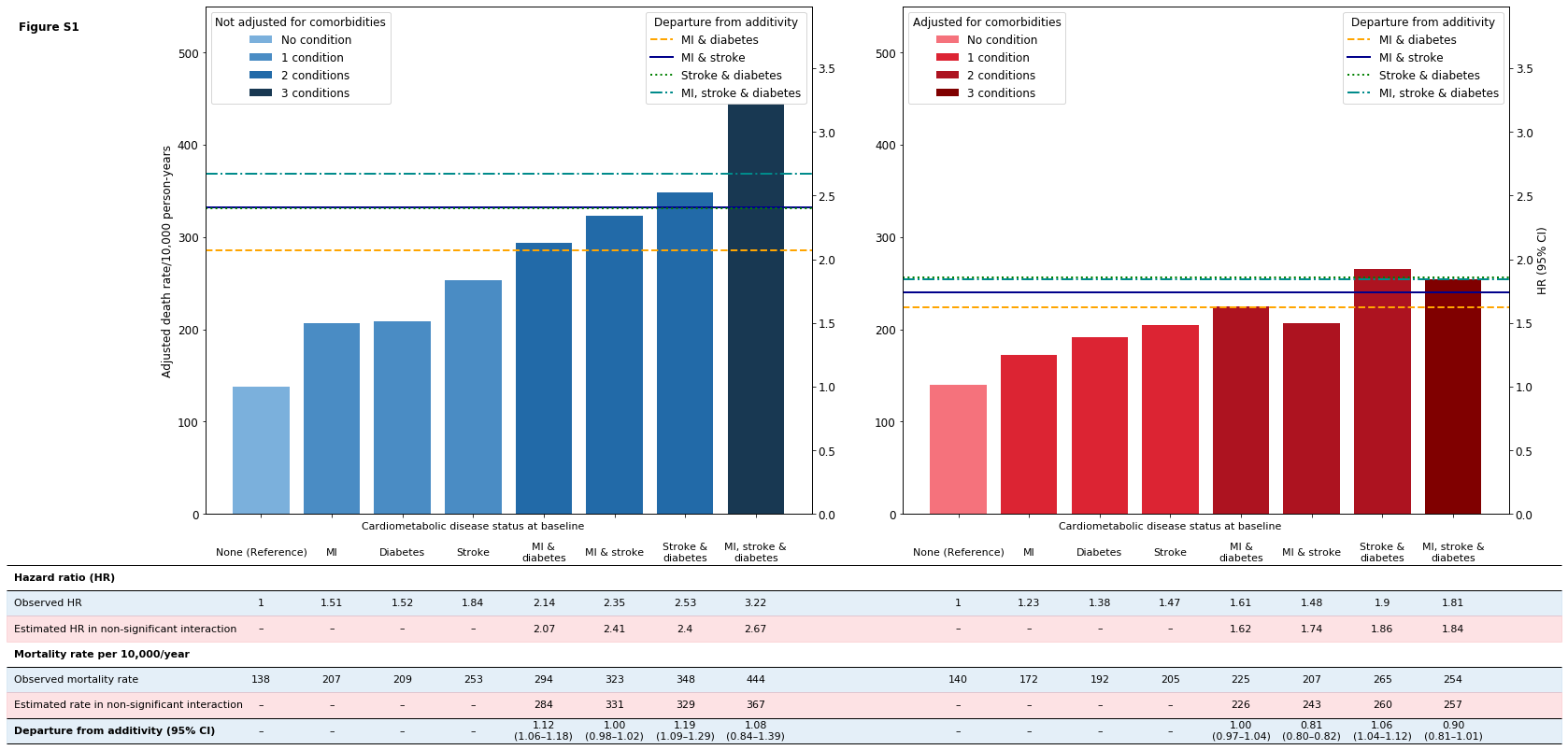


**Mapping of Read codes to chronic conditions**

| **Condition** | **Code** |
| --- | --- |
| adjustment disorder | F43 |
| affective disorder | F39 |
| affective disorder | F38 |
| affective disorder | F34 |
| anaemia | P614 |
| anaemia | P613 |
| anaemia | P612 |
| anaemia | P589 |
| anaemia | P588 |
| anaemia | P569 |
| anaemia | P559 |
| anaemia | P558 |
| anaemia | O990 |
| anaemia | D64 |
| anaemia | D63 |
| anaemia | D61 |
| anaemia | D60 |
| anaemia | D59 |
| anaemia | D58 |
| anaemia | D57 |
| anaemia | D569 |
| anaemia | D568 |
| anaemia | D563 |
| anaemia | D562 |
| anaemia | D561 |
| anaemia | D560 |
| anaemia | D55 |
| anaemia | D53 |
| anaemia | D52 |
| anaemia | D51 |
| anaemia | D50 |
| anaemia | D46 |
| anxiety | F41 |
| anxiety | F40 |
| arthropathy | M48.99 |
| arthropathy | M48.98 |
| arthropathy | M48.97 |
| arthropathy | M48.96 |
| arthropathy | M48.95 |
| arthropathy | M48.94 |
| arthropathy | M48.93 |
| **Condition** | **Code** |
| arthropathy | M48.92 |
| arthropathy | M48.91 |
| arthropathy | M48.90 |
| arthropathy | M48.9 |
| arthropathy | M48.89 |
| arthropathy | M48.88 |
| arthropathy | M48.87 |
| arthropathy | M48.86 |
| arthropathy | M48.85 |
| arthropathy | M48.84 |
| arthropathy | M48.83 |
| arthropathy | M48.82 |
| arthropathy | M48.81 |
| arthropathy | M48.80 |
| arthropathy | M48.8 |
| arthropathy | M48.59 |
| arthropathy | M48.58 |
| arthropathy | M48.57 |
| arthropathy | M48.56 |
| arthropathy | M48.55 |
| arthropathy | M48.54 |
| arthropathy | M48.53 |
| arthropathy | M48.52 |
| arthropathy | M48.51 |
| arthropathy | M48.50 |
| arthropathy | M48.5 |
| arthropathy | M48.49 |
| arthropathy | M48.48 |
| arthropathy | M48.47 |
| arthropathy | M48.46 |
| arthropathy | M48.45 |
| arthropathy | M48.44 |
| arthropathy | M48.43 |
| arthropathy | M48.42 |
| arthropathy | M48.41 |
| arthropathy | M48.40 |
| arthropathy | M48.4 |
| arthropathy | M48.39 |
| arthropathy | M48.38 |
| arthropathy | M48.37 |
| arthropathy | M48.36 |
| arthropathy | M48.35 |
| arthropathy | M48.34 |
| arthropathy | M48.33 |
| arthropathy | M48.32 |
| arthropathy | M48.31 |
| arthropathy | M48.30 |
| arthropathy | M48.3 |
| arthropathy | M48.29 |
| arthropathy | M48.28 |
| arthropathy | M48.27 |
| arthropathy | M48.26 |
| arthropathy | M48.25 |
| arthropathy | M48.24 |
| arthropathy | M48.23 |
| arthropathy | M48.22 |
| arthropathy | M48.21 |
| arthropathy | M48.20 |
| arthropathy | M48.2 |
| arthropathy | M48.19 |
| arthropathy | M48.18 |
| arthropathy | M48.17 |
| arthropathy | M48.16 |
| arthropathy | M48.15 |
| arthropathy | M48.14 |
| arthropathy | M48.13 |
| arthropathy | M48.12 |
| arthropathy | M48.11 |
| arthropathy | M48.10 |
| arthropathy | M48.1 |
| arthropathy | M48.09 |
| arthropathy | M48.08 |
| arthropathy | M48.07 |
| arthropathy | M48.06 |
| arthropathy | M48.05 |
| arthropathy | M48.04 |
| arthropathy | M48.03 |
| arthropathy | M48.02 |
| arthropathy | M48.01 |
| arthropathy | M48.00 |
| arthropathy | M48.0 |
| arthropathy | M48 |
| arthropathy | M479 |
| arthropathy | M478 |
| arthropathy | M472 |
| arthropathy | M471 |
| arthropathy | M47.99 |
| arthropathy | M47.98 |
| arthropathy | M47.97 |
| arthropathy | M47.96 |
| arthropathy | M47.95 |
| arthropathy | M47.94 |
| arthropathy | M47.93 |
| arthropathy | M47.92 |
| arthropathy | M47.91 |
| arthropathy | M47.90 |
| arthropathy | M47.9 |
| arthropathy | M47.89 |
| arthropathy | M47.88 |
| arthropathy | M47.87 |
| arthropathy | M47.86 |
| arthropathy | M47.85 |
| arthropathy | M47.84 |
| arthropathy | M47.83 |
| arthropathy | M47.82 |
| arthropathy | M47.81 |
| arthropathy | M47.80 |
| arthropathy | M47.8 |
| arthropathy | M47.29 |
| arthropathy | M47.28 |
| arthropathy | M47.27 |
| arthropathy | M47.26 |
| arthropathy | M47.25 |
| arthropathy | M47.24 |
| arthropathy | M47.23 |
| arthropathy | M47.22 |
| arthropathy | M47.21 |
| arthropathy | M47.20 |
| arthropathy | M47.2 |
| arthropathy | M47.19 |
| arthropathy | M47.18 |
| arthropathy | M47.17 |
| arthropathy | M47.16 |
| arthropathy | M47.15 |
| arthropathy | M47.14 |
| arthropathy | M47.13 |
| arthropathy | M47.12 |
| arthropathy | M47.11 |
| arthropathy | M47.10 |
| arthropathy | M47.1 |
| arthropathy | M47.09 |
| arthropathy | M47.03 |
| arthropathy | M47.02 |
| arthropathy | M47.01 |
| arthropathy | M47.00 |
| arthropathy | M47.0 |
| arthropathy | M47 |
| arthropathy | M46.99 |
| arthropathy | M46.98 |
| arthropathy | M46.97 |
| arthropathy | M46.96 |
| arthropathy | M46.95 |
| arthropathy | M46.94 |
| arthropathy | M46.93 |
| arthropathy | M46.92 |
| arthropathy | M46.91 |
| arthropathy | M46.90 |
| arthropathy | M46.9 |
| arthropathy | M46.89 |
| arthropathy | M46.88 |
| arthropathy | M46.87 |
| arthropathy | M46.86 |
| arthropathy | M46.85 |
| arthropathy | M46.84 |
| arthropathy | M46.83 |
| arthropathy | M46.82 |
| arthropathy | M46.81 |
| arthropathy | M46.80 |
| arthropathy | M46.8 |
| arthropathy | M46.59 |
| arthropathy | M46.58 |
| arthropathy | M46.57 |
| arthropathy | M46.56 |
| arthropathy | M46.55 |
| arthropathy | M46.54 |
| arthropathy | M46.53 |
| arthropathy | M46.52 |
| arthropathy | M46.51 |
| arthropathy | M46.50 |
| arthropathy | M46.5 |
| arthropathy | M46.49 |
| arthropathy | M46.48 |
| arthropathy | M46.47 |
| arthropathy | M46.46 |
| arthropathy | M46.45 |
| arthropathy | M46.44 |
| arthropathy | M46.43 |
| arthropathy | M46.42 |
| arthropathy | M46.41 |
| arthropathy | M46.40 |
| arthropathy | M46.4 |
| arthropathy | M46.39 |
| arthropathy | M46.38 |
| arthropathy | M46.37 |
| arthropathy | M46.36 |
| arthropathy | M46.35 |
| arthropathy | M46.34 |
| arthropathy | M46.33 |
| arthropathy | M46.32 |
| arthropathy | M46.31 |
| arthropathy | M46.30 |
| arthropathy | M46.3 |
| arthropathy | M46.29 |
| arthropathy | M46.28 |
| arthropathy | M46.27 |
| arthropathy | M46.26 |
| arthropathy | M46.25 |
| arthropathy | M46.24 |
| arthropathy | M46.23 |
| arthropathy | M46.22 |
| arthropathy | M46.21 |
| arthropathy | M46.20 |
| arthropathy | M46.2 |
| arthropathy | M46.18 |
| arthropathy | M46.17 |
| arthropathy | M46.1 |
| arthropathy | M46.09 |
| arthropathy | M46.08 |
| arthropathy | M46.07 |
| arthropathy | M46.06 |
| arthropathy | M46.05 |
| arthropathy | M46.04 |
| arthropathy | M46.03 |
| arthropathy | M46.02 |
| arthropathy | M46.01 |
| arthropathy | M46.00 |
| arthropathy | M46.0 |
| arthropathy | M46 |
| arthropathy | M45.X9 |
| arthropathy | M45.X8 |
| arthropathy | M45.X7 |
| arthropathy | M45.X6 |
| arthropathy | M45.X5 |
| arthropathy | M45.X4 |
| arthropathy | M45.X3 |
| arthropathy | M45.X2 |
| arthropathy | M45.X1 |
| arthropathy | M45.X0 |
| arthropathy | M45 |
| arthropathy | M43.99 |
| arthropathy | M43.98 |
| arthropathy | M43.97 |
| arthropathy | M43.96 |
| arthropathy | M43.95 |
| arthropathy | M43.94 |
| arthropathy | M43.93 |
| arthropathy | M43.92 |
| arthropathy | M43.91 |
| arthropathy | M43.90 |
| arthropathy | M43.9 |
| arthropathy | M43.89 |
| arthropathy | M43.88 |
| arthropathy | M43.87 |
| arthropathy | M43.86 |
| arthropathy | M43.85 |
| arthropathy | M43.84 |
| arthropathy | M43.83 |
| arthropathy | M43.82 |
| arthropathy | M43.81 |
| arthropathy | M43.80 |
| arthropathy | M43.8 |
| arthropathy | M43.63 |
| arthropathy | M43.62 |
| arthropathy | M43.61 |
| arthropathy | M43.60 |
| arthropathy | M43.6 |
| arthropathy | M43.59 |
| arthropathy | M43.58 |
| arthropathy | M43.57 |
| arthropathy | M43.56 |
| arthropathy | M43.55 |
| arthropathy | M43.54 |
| arthropathy | M43.53 |
| arthropathy | M43.52 |
| arthropathy | M43.51 |
| arthropathy | M43.50 |
| arthropathy | M43.5 |
| arthropathy | M43.41 |
| arthropathy | M43.4 |
| arthropathy | M43.31 |
| arthropathy | M43.3 |
| arthropathy | M43.29 |
| arthropathy | M43.28 |
| arthropathy | M43.27 |
| arthropathy | M43.26 |
| arthropathy | M43.25 |
| arthropathy | M43.24 |
| arthropathy | M43.23 |
| arthropathy | M43.22 |
| arthropathy | M43.21 |
| arthropathy | M43.20 |
| arthropathy | M43.2 |
| arthropathy | M43.19 |
| arthropathy | M43.18 |
| arthropathy | M43.17 |
| arthropathy | M43.16 |
| arthropathy | M43.15 |
| arthropathy | M43.14 |
| arthropathy | M43.13 |
| arthropathy | M43.12 |
| arthropathy | M43.11 |
| arthropathy | M43.10 |
| arthropathy | M43.1 |
| arthropathy | M43.09 |
| arthropathy | M43.08 |
| arthropathy | M43.07 |
| arthropathy | M43.06 |
| arthropathy | M43.05 |
| arthropathy | M43.04 |
| arthropathy | M43.03 |
| arthropathy | M43.02 |
| arthropathy | M43.01 |
| arthropathy | M43.00 |
| arthropathy | M43.0 |
| arthropathy | M43 |
| arthropathy | M42.99 |
| arthropathy | M42.98 |
| arthropathy | M42.97 |
| arthropathy | M42.96 |
| arthropathy | M42.95 |
| arthropathy | M42.94 |
| arthropathy | M42.93 |
| arthropathy | M42.92 |
| arthropathy | M42.91 |
| arthropathy | M42.90 |
| arthropathy | M42.9 |
| arthropathy | M42.19 |
| arthropathy | M42.18 |
| arthropathy | M42.17 |
| arthropathy | M42.16 |
| arthropathy | M42.15 |
| arthropathy | M42.14 |
| arthropathy | M42.13 |
| arthropathy | M42.12 |
| arthropathy | M42.11 |
| arthropathy | M42.10 |
| arthropathy | M42.1 |
| arthropathy | M42.09 |
| arthropathy | M42.08 |
| arthropathy | M42.07 |
| arthropathy | M42.06 |
| arthropathy | M42.05 |
| arthropathy | M42.04 |
| arthropathy | M42.03 |
| arthropathy | M42.02 |
| arthropathy | M42.01 |
| arthropathy | M42.00 |
| arthropathy | M42.0 |
| arthropathy | M42 |
| arthropathy | M41.99 |
| arthropathy | M41.98 |
| arthropathy | M41.97 |
| arthropathy | M41.96 |
| arthropathy | M41.95 |
| arthropathy | M41.94 |
| arthropathy | M41.93 |
| arthropathy | M41.92 |
| arthropathy | M41.91 |
| arthropathy | M41.90 |
| arthropathy | M41.9 |
| arthropathy | M41.89 |
| arthropathy | M41.88 |
| arthropathy | M41.87 |
| arthropathy | M41.86 |
| arthropathy | M41.85 |
| arthropathy | M41.84 |
| arthropathy | M41.83 |
| arthropathy | M41.82 |
| arthropathy | M41.81 |
| arthropathy | M41.80 |
| arthropathy | M41.8 |
| arthropathy | M41.59 |
| arthropathy | M41.58 |
| arthropathy | M41.57 |
| arthropathy | M41.56 |
| arthropathy | M41.55 |
| arthropathy | M41.54 |
| arthropathy | M41.53 |
| arthropathy | M41.52 |
| arthropathy | M41.51 |
| arthropathy | M41.50 |
| arthropathy | M41.5 |
| arthropathy | M41.49 |
| arthropathy | M41.48 |
| arthropathy | M41.47 |
| arthropathy | M41.46 |
| arthropathy | M41.45 |
| arthropathy | M41.44 |
| arthropathy | M41.43 |
| arthropathy | M41.42 |
| arthropathy | M41.41 |
| arthropathy | M41.40 |
| arthropathy | M41.4 |
| arthropathy | M41.35 |
| arthropathy | M41.34 |
| arthropathy | M41.30 |
| arthropathy | M41.3 |
| arthropathy | M41.29 |
| arthropathy | M41.28 |
| arthropathy | M41.27 |
| arthropathy | M41.26 |
| arthropathy | M41.25 |
| arthropathy | M41.24 |
| arthropathy | M41.23 |
| arthropathy | M41.22 |
| arthropathy | M41.21 |
| arthropathy | M41.20 |
| arthropathy | M41.2 |
| arthropathy | M41.19 |
| arthropathy | M41.18 |
| arthropathy | M41.17 |
| arthropathy | M41.16 |
| arthropathy | M41.15 |
| arthropathy | M41.14 |
| arthropathy | M41.13 |
| arthropathy | M41.12 |
| arthropathy | M41.11 |
| arthropathy | M41.10 |
| arthropathy | M41.1 |
| arthropathy | M41.09 |
| arthropathy | M41.08 |
| arthropathy | M41.07 |
| arthropathy | M41.06 |
| arthropathy | M41.05 |
| arthropathy | M41.04 |
| arthropathy | M41.03 |
| arthropathy | M41.02 |
| arthropathy | M41.01 |
| arthropathy | M41.00 |
| arthropathy | M41.0 |
| arthropathy | M41 |
| arthropathy | M40.59 |
| arthropathy | M40.58 |
| arthropathy | M40.57 |
| arthropathy | M40.56 |
| arthropathy | M40.55 |
| arthropathy | M40.54 |
| arthropathy | M40.53 |
| arthropathy | M40.52 |
| arthropathy | M40.51 |
| arthropathy | M40.50 |
| arthropathy | M40.5 |
| arthropathy | M40.49 |
| arthropathy | M40.48 |
| arthropathy | M40.47 |
| arthropathy | M40.46 |
| arthropathy | M40.45 |
| arthropathy | M40.44 |
| arthropathy | M40.43 |
| arthropathy | M40.42 |
| arthropathy | M40.41 |
| arthropathy | M40.40 |
| arthropathy | M40.4 |
| arthropathy | M40.39 |
| arthropathy | M40.38 |
| arthropathy | M40.37 |
| arthropathy | M40.36 |
| arthropathy | M40.35 |
| arthropathy | M40.34 |
| arthropathy | M40.33 |
| arthropathy | M40.32 |
| arthropathy | M40.31 |
| arthropathy | M40.30 |
| arthropathy | M40.3 |
| arthropathy | M40.29 |
| arthropathy | M40.28 |
| arthropathy | M40.27 |
| arthropathy | M40.26 |
| arthropathy | M40.25 |
| arthropathy | M40.24 |
| arthropathy | M40.23 |
| arthropathy | M40.22 |
| arthropathy | M40.21 |
| arthropathy | M40.20 |
| arthropathy | M40.2 |
| arthropathy | M40.19 |
| arthropathy | M40.18 |
| arthropathy | M40.17 |
| arthropathy | M40.16 |
| arthropathy | M40.15 |
| arthropathy | M40.14 |
| arthropathy | M40.13 |
| arthropathy | M40.12 |
| arthropathy | M40.11 |
| arthropathy | M40.10 |
| arthropathy | M40.1 |
| arthropathy | M40.09 |
| arthropathy | M40.08 |
| arthropathy | M40.07 |
| arthropathy | M40.06 |
| arthropathy | M40.05 |
| arthropathy | M40.04 |
| arthropathy | M40.03 |
| arthropathy | M40.02 |
| arthropathy | M40.01 |
| arthropathy | M40.00 |
| arthropathy | M40.0 |
| arthropathy | M40 |
| arthropathy | M25.99 |
| arthropathy | M25.98 |
| arthropathy | M25.97 |
| arthropathy | M25.96 |
| arthropathy | M25.95 |
| arthropathy | M25.94 |
| arthropathy | M25.93 |
| arthropathy | M25.92 |
| arthropathy | M25.91 |
| arthropathy | M25.90 |
| arthropathy | M25.9 |
| arthropathy | M25.89 |
| arthropathy | M25.88 |
| arthropathy | M25.87 |
| arthropathy | M25.86 |
| arthropathy | M25.85 |
| arthropathy | M25.84 |
| arthropathy | M25.83 |
| arthropathy | M25.82 |
| arthropathy | M25.81 |
| arthropathy | M25.80 |
| arthropathy | M25.8 |
| arthropathy | M25.79 |
| arthropathy | M25.78 |
| arthropathy | M25.77 |
| arthropathy | M25.76 |
| arthropathy | M25.75 |
| arthropathy | M25.74 |
| arthropathy | M25.73 |
| arthropathy | M25.72 |
| arthropathy | M25.71 |
| arthropathy | M25.70 |
| arthropathy | M25.7 |
| arthropathy | M25.69 |
| arthropathy | M25.68 |
| arthropathy | M25.67 |
| arthropathy | M25.66 |
| arthropathy | M25.65 |
| arthropathy | M25.64 |
| arthropathy | M25.63 |
| arthropathy | M25.62 |
| arthropathy | M25.61 |
| arthropathy | M25.60 |
| arthropathy | M25.6 |
| arthropathy | M25.59 |
| arthropathy | M25.58 |
| arthropathy | M25.57 |
| arthropathy | M25.56 |
| arthropathy | M25.55 |
| arthropathy | M25.54 |
| arthropathy | M25.53 |
| arthropathy | M25.52 |
| arthropathy | M25.51 |
| arthropathy | M25.50 |
| arthropathy | M25.5 |
| arthropathy | M25 |
| arthropathy | M19.99 |
| arthropathy | M19.98 |
| arthropathy | M19.97 |
| arthropathy | M19.96 |
| arthropathy | M19.95 |
| arthropathy | M19.94 |
| arthropathy | M19.93 |
| arthropathy | M19.92 |
| arthropathy | M19.91 |
| arthropathy | M19.90 |
| arthropathy | M19.9 |
| arthropathy | M19.89 |
| arthropathy | M19.88 |
| arthropathy | M19.87 |
| arthropathy | M19.86 |
| arthropathy | M19.85 |
| arthropathy | M19.84 |
| arthropathy | M19.83 |
| arthropathy | M19.82 |
| arthropathy | M19.81 |
| arthropathy | M19.80 |
| arthropathy | M19.8 |
| arthropathy | M19.29 |
| arthropathy | M19.28 |
| arthropathy | M19.27 |
| arthropathy | M19.26 |
| arthropathy | M19.25 |
| arthropathy | M19.24 |
| arthropathy | M19.23 |
| arthropathy | M19.22 |
| arthropathy | M19.21 |
| arthropathy | M19.20 |
| arthropathy | M19.2 |
| arthropathy | M19.19 |
| arthropathy | M19.18 |
| arthropathy | M19.17 |
| arthropathy | M19.16 |
| arthropathy | M19.15 |
| arthropathy | M19.14 |
| arthropathy | M19.13 |
| arthropathy | M19.12 |
| arthropathy | M19.11 |
| arthropathy | M19.10 |
| arthropathy | M19.1 |
| arthropathy | M19.09 |
| arthropathy | M19.08 |
| arthropathy | M19.07 |
| arthropathy | M19.06 |
| arthropathy | M19.05 |
| arthropathy | M19.04 |
| arthropathy | M19.03 |
| arthropathy | M19.02 |
| arthropathy | M19.01 |
| arthropathy | M19.00 |
| arthropathy | M19.0 |
| arthropathy | M19 |
| arthropathy | M18.9 |
| arthropathy | M18.5 |
| arthropathy | M18.4 |
| arthropathy | M18.3 |
| arthropathy | M18.2 |
| arthropathy | M18.1 |
| arthropathy | M18.0 |
| arthropathy | M18 |
| arthropathy | M17.9 |
| arthropathy | M17.5 |
| arthropathy | M17.4 |
| arthropathy | M17.3 |
| arthropathy | M17.2 |
| arthropathy | M17.1 |
| arthropathy | M17.0 |
| arthropathy | M17 |
| arthropathy | M16.9 |
| arthropathy | M16.7 |
| arthropathy | M16.6 |
| arthropathy | M16.5 |
| arthropathy | M16.4 |
| arthropathy | M16.3 |
| arthropathy | M16.2 |
| arthropathy | M16.1 |
| arthropathy | M16.0 |
| arthropathy | M16 |
| arthropathy | M15.9 |
| arthropathy | M15.8 |
| arthropathy | M15.4 |
| arthropathy | M15.3 |
| arthropathy | M15.2 |
| arthropathy | M15.1 |
| arthropathy | M15.0 |
| arthropathy | M15 |
| arthropathy | M14.8 |
| arthropathy | M14.6 |
| arthropathy | M14.5 |
| arthropathy | M14.4 |
| arthropathy | M14.3 |
| arthropathy | M14.2 |
| arthropathy | M14.1 |
| arthropathy | M14 |
| arthropathy | M13.99 |
| arthropathy | M13.98 |
| arthropathy | M13.97 |
| arthropathy | M13.96 |
| arthropathy | M13.95 |
| arthropathy | M13.94 |
| arthropathy | M13.93 |
| arthropathy | M13.92 |
| arthropathy | M13.91 |
| arthropathy | M13.90 |
| arthropathy | M13.9 |
| arthropathy | M13.89 |
| arthropathy | M13.88 |
| arthropathy | M13.87 |
| arthropathy | M13.86 |
| arthropathy | M13.85 |
| arthropathy | M13.84 |
| arthropathy | M13.83 |
| arthropathy | M13.82 |
| arthropathy | M13.81 |
| arthropathy | M13.80 |
| arthropathy | M13.8 |
| arthropathy | M13.19 |
| arthropathy | M13.18 |
| arthropathy | M13.17 |
| arthropathy | M13.16 |
| arthropathy | M13.15 |
| arthropathy | M13.14 |
| arthropathy | M13.13 |
| arthropathy | M13.12 |
| arthropathy | M13.11 |
| arthropathy | M13.10 |
| arthropathy | M13.1 |
| arthropathy | M13.09 |
| arthropathy | M13.08 |
| arthropathy | M13.07 |
| arthropathy | M13.06 |
| arthropathy | M13.05 |
| arthropathy | M13.04 |
| arthropathy | M13.03 |
| arthropathy | M13.02 |
| arthropathy | M13.01 |
| arthropathy | M13.00 |
| arthropathy | M13.0 |
| arthropathy | M13 |
| arthropathy | M12.89 |
| arthropathy | M12.88 |
| arthropathy | M12.87 |
| arthropathy | M12.86 |
| arthropathy | M12.85 |
| arthropathy | M12.84 |
| arthropathy | M12.83 |
| arthropathy | M12.82 |
| arthropathy | M12.81 |
| arthropathy | M12.80 |
| arthropathy | M12.8 |
| arthropathy | M12.59 |
| arthropathy | M12.58 |
| arthropathy | M12.57 |
| arthropathy | M12.56 |
| arthropathy | M12.55 |
| arthropathy | M12.54 |
| arthropathy | M12.53 |
| arthropathy | M12.52 |
| arthropathy | M12.51 |
| arthropathy | M12.50 |
| arthropathy | M12.5 |
| arthropathy | M12.49 |
| arthropathy | M12.48 |
| arthropathy | M12.47 |
| arthropathy | M12.46 |
| arthropathy | M12.45 |
| arthropathy | M12.44 |
| arthropathy | M12.43 |
| arthropathy | M12.42 |
| arthropathy | M12.41 |
| arthropathy | M12.40 |
| arthropathy | M12.4 |
| arthropathy | M12.39 |
| arthropathy | M12.38 |
| arthropathy | M12.37 |
| arthropathy | M12.36 |
| arthropathy | M12.35 |
| arthropathy | M12.34 |
| arthropathy | M12.33 |
| arthropathy | M12.32 |
| arthropathy | M12.31 |
| arthropathy | M12.30 |
| arthropathy | M12.3 |
| arthropathy | M12.29 |
| arthropathy | M12.28 |
| arthropathy | M12.27 |
| arthropathy | M12.26 |
| arthropathy | M12.25 |
| arthropathy | M12.24 |
| arthropathy | M12.23 |
| arthropathy | M12.22 |
| arthropathy | M12.21 |
| arthropathy | M12.20 |
| arthropathy | M12.2 |
| arthropathy | M12.19 |
| arthropathy | M12.18 |
| arthropathy | M12.17 |
| arthropathy | M12.16 |
| arthropathy | M12.15 |
| arthropathy | M12.14 |
| arthropathy | M12.13 |
| arthropathy | M12.12 |
| arthropathy | M12.11 |
| arthropathy | M12.10 |
| arthropathy | M12.1 |
| arthropathy | M12.09 |
| arthropathy | M12.08 |
| arthropathy | M12.07 |
| arthropathy | M12.06 |
| arthropathy | M12.05 |
| arthropathy | M12.04 |
| arthropathy | M12.03 |
| arthropathy | M12.02 |
| arthropathy | M12.01 |
| arthropathy | M12.00 |
| arthropathy | M12.0 |
| arthropathy | M12 |
| arthropathy | M11.99 |
| arthropathy | M11.98 |
| arthropathy | M11.97 |
| arthropathy | M11.96 |
| arthropathy | M11.95 |
| arthropathy | M11.94 |
| arthropathy | M11.93 |
| arthropathy | M11.92 |
| arthropathy | M11.91 |
| arthropathy | M11.90 |
| arthropathy | M11.9 |
| arthropathy | M11.89 |
| arthropathy | M11.88 |
| arthropathy | M11.87 |
| arthropathy | M11.86 |
| arthropathy | M11.85 |
| arthropathy | M11.84 |
| arthropathy | M11.83 |
| arthropathy | M11.82 |
| arthropathy | M11.81 |
| arthropathy | M11.80 |
| arthropathy | M11.8 |
| arthropathy | M11.29 |
| arthropathy | M11.28 |
| arthropathy | M11.27 |
| arthropathy | M11.26 |
| arthropathy | M11.25 |
| arthropathy | M11.24 |
| arthropathy | M11.23 |
| arthropathy | M11.22 |
| arthropathy | M11.21 |
| arthropathy | M11.20 |
| arthropathy | M11.2 |
| arthropathy | M11.19 |
| arthropathy | M11.18 |
| arthropathy | M11.17 |
| arthropathy | M11.16 |
| arthropathy | M11.15 |
| arthropathy | M11.14 |
| arthropathy | M11.13 |
| arthropathy | M11.12 |
| arthropathy | M11.11 |
| arthropathy | M11.10 |
| arthropathy | M11.1 |
| arthropathy | M11.09 |
| arthropathy | M11.08 |
| arthropathy | M11.07 |
| arthropathy | M11.06 |
| arthropathy | M11.05 |
| arthropathy | M11.04 |
| arthropathy | M11.03 |
| arthropathy | M11.02 |
| arthropathy | M11.01 |
| arthropathy | M11.00 |
| arthropathy | M11.0 |
| arthropathy | M11 |
| arthropathy | M07.69 |
| arthropathy | M07.68 |
| arthropathy | M07.67 |
| arthropathy | M07.66 |
| arthropathy | M07.65 |
| arthropathy | M07.64 |
| arthropathy | M07.63 |
| arthropathy | M07.62 |
| arthropathy | M07.61 |
| arthropathy | M07.60 |
| arthropathy | M07.6 |
| arthropathy | M07.59 |
| arthropathy | M07.58 |
| arthropathy | M07.57 |
| arthropathy | M07.56 |
| arthropathy | M07.55 |
| arthropathy | M07.54 |
| arthropathy | M07.53 |
| arthropathy | M07.52 |
| arthropathy | M07.51 |
| arthropathy | M07.50 |
| arthropathy | M07.5 |
| arthropathy | M07.49 |
| arthropathy | M07.48 |
| arthropathy | M07.47 |
| arthropathy | M07.46 |
| arthropathy | M07.45 |
| arthropathy | M07.44 |
| arthropathy | M07.43 |
| arthropathy | M07.42 |
| arthropathy | M07.41 |
| arthropathy | M07.40 |
| arthropathy | M07.4 |
| arthropathy | M07.39 |
| arthropathy | M07.38 |
| arthropathy | M07.37 |
| arthropathy | M07.36 |
| arthropathy | M07.35 |
| arthropathy | M07.34 |
| arthropathy | M07.33 |
| arthropathy | M07.32 |
| arthropathy | M07.31 |
| arthropathy | M07.30 |
| arthropathy | M07.3 |
| arthropathy | M07.29 |
| arthropathy | M07.28* |
| arthropathy | M07.27 |
| arthropathy | M07.26 |
| arthropathy | M07.25 |
| arthropathy | M07.24 |
| arthropathy | M07.23 |
| arthropathy | M07.22 |
| arthropathy | M07.21 |
| arthropathy | M07.20 |
| arthropathy | M07.2 |
| arthropathy | M07.19 |
| arthropathy | M07.18 |
| arthropathy | M07.17 |
| arthropathy | M07.16 |
| arthropathy | M07.15 |
| arthropathy | M07.14 |
| arthropathy | M07.13 |
| arthropathy | M07.12 |
| arthropathy | M07.11 |
| arthropathy | M07.10 |
| arthropathy | M07.1 |
| arthropathy | M07.09 |
| arthropathy | M07.07 |
| arthropathy | M07.04 |
| arthropathy | M07.00 |
| arthropathy | M07.0 |
| arthropathy | M07 |
| arthropathy | M06.49 |
| arthropathy | M06.48 |
| arthropathy | M06.47 |
| arthropathy | M06.46 |
| arthropathy | M06.45 |
| arthropathy | M06.44 |
| arthropathy | M06.43 |
| arthropathy | M06.42 |
| arthropathy | M06.41 |
| arthropathy | M06.40 |
| arthropathy | M06.4 |
| arthropathy | M06.19 |
| arthropathy | M06.18 |
| arthropathy | M06.17 |
| arthropathy | M06.16 |
| arthropathy | M06.15 |
| arthropathy | M06.14 |
| arthropathy | M06.13 |
| arthropathy | M06.12 |
| arthropathy | M06.11 |
| arthropathy | M06.10 |
| arthropathy | M06.1 |
| asthma | J82 |
| asthma | J46 |
| asthma | J45.9 |
| asthma | J45.8 |
| asthma | J45.1 |
| asthma | J45.0 |
| asthma | J45 |
| asthma | J44.9 |
| asthma | J44.8 |
| bipolar disorder | F31 |
| bipolar disorder | F30 |
| bone disease (osteoporosis) | M82* |
| bone disease (osteoporosis) | M81 |
| bone disease (osteoporosis) | M80 |
| cardiac arrhythmia | X504 |
| cardiac arrhythmia | X502 |
| cardiac arrhythmia | X501 |
| cardiac arrhythmia | K624 |
| cardiac arrhythmia | K623 |
| cardiac arrhythmia | K622 |
| cardiac arrhythmia | K621 |
| cardiac arrhythmia | K62 |
| cardiac arrhythmia | K575 |
| cardiac arrhythmia | K571 |
| cardiac arrhythmia | K521 |
| cardiac arrhythmia | I49 |
| cardiac arrhythmia | I48 |
| cardiac arrhythmia | I47 |
| cardiac arrhythmia | I45 |
| cardiac arrhythmia | I44 |
| chronic kidney disease | Z992 |
| chronic kidney disease | Z940 |
| chronic kidney disease | Z49 |
| chronic kidney disease | Y841 |
| chronic kidney disease | X421 |
| chronic kidney disease | X412 |
| chronic kidney disease | X411 |
| chronic kidney disease | X406 |
| chronic kidney disease | X405 |
| chronic kidney disease | X403 |
| chronic kidney disease | X402 |
| chronic kidney disease | X401 |
| chronic kidney disease | T861 |
| chronic kidney disease | N25 |
| chronic kidney disease | N19 |
| chronic kidney disease | N189 |
| chronic kidney disease | N185 |
| chronic kidney disease | N184 |
| chronic kidney disease | N183 |
| chronic kidney disease | N182 |
| chronic kidney disease | N181 |
| chronic kidney disease | N17 |
| chronic kidney disease | N16* |
| chronic kidney disease | N15 |
| chronic kidney disease | N14 |
| chronic kidney disease | N13 |
| chronic kidney disease | N12 |
| chronic kidney disease | N11 |
| chronic kidney disease | N10 |
| chronic kidney disease | N08* |
| chronic kidney disease | N074 |
| chronic kidney disease | N073 |
| chronic kidney disease | N072 |
| chronic kidney disease | N07 |
| chronic kidney disease | N056 |
| chronic kidney disease | N055 |
| chronic kidney disease | N054 |
| chronic kidney disease | N053 |
| chronic kidney disease | N052 |
| chronic kidney disease | N05 |
| chronic kidney disease | N04 |
| chronic kidney disease | N03 |
| chronic kidney disease | N01 |
| chronic kidney disease | N00 |
| chronic kidney disease | L746 |
| chronic obstructive pulmonary disease | J449 |
| chronic obstructive pulmonary disease | J448 |
| chronic obstructive pulmonary disease | J441 |
| chronic obstructive pulmonary disease | J440 |
| chronic obstructive pulmonary disease | J43 |
| chronic obstructive pulmonary disease | J42 |
| chronic obstructive pulmonary disease | J41 |
| chronic obstructive pulmonary disease | J40 |
| chronic obstructive pulmonary disease | J20 |
| connective tissue disease | M36.8 |
| connective tissue disease | M36.4 |
| connective tissue disease | M36.3 |
| connective tissue disease | M36.2 |
| connective tissue disease | M36.1 |
| connective tissue disease | M36.0 |
| connective tissue disease | M36 |
| connective tissue disease | M35.9 |
| connective tissue disease | M35.8 |
| connective tissue disease | M35.7 |
| connective tissue disease | M35.6 |
| connective tissue disease | M35.5 |
| connective tissue disease | M35.4 |
| connective tissue disease | M35.3 |
| connective tissue disease | M35.3 |
| connective tissue disease | M35.2 |
| connective tissue disease | M35.1 |
| connective tissue disease | M35.0 |
| connective tissue disease | M35 |
| connective tissue disease | M34.9 |
| connective tissue disease | M34.8 |
| connective tissue disease | M34.2 |
| connective tissue disease | M34.1 |
| connective tissue disease | M34.0 |
| connective tissue disease | M34 |
| connective tissue disease | M33.9 |
| connective tissue disease | M33.9 |
| connective tissue disease | M33.2 |
| connective tissue disease | M33.2 |
| connective tissue disease | M33.1 |
| connective tissue disease | M33.1 |
| connective tissue disease | M33.0 |
| connective tissue disease | M33.0 |
| connective tissue disease | M33 |
| connective tissue disease | M32.9 |
| connective tissue disease | M32.8 |
| connective tissue disease | M32.1 |
| connective tissue disease | M32.0 |
| connective tissue disease | M32 |
| connective tissue disease | M31.9 |
| connective tissue disease | M31.8 |
| connective tissue disease | M31.7 |
| connective tissue disease | M31.6 |
| connective tissue disease | M31.5 |
| connective tissue disease | M31.4 |
| connective tissue disease | M31.3 |
| connective tissue disease | M31.2 |
| connective tissue disease | M31.1 |
| connective tissue disease | M31.0 |
| connective tissue disease | M31 |
| connective tissue disease | M30.8 |
| connective tissue disease | M30.3 |
| connective tissue disease | M30.2 |
| connective tissue disease | M30.1 |
| connective tissue disease | M30.0 |
| connective tissue disease | M30 |
| dementia | G30 |
| dementia | F051 |
| dementia | F03 |
| dementia | F02 |
| dementia | F01 |
| dementia | F00 |
| depression | F381 |
| depression | F341 |
| depression | F339 |
| depression | F338 |
| depression | F334 |
| depression | F333 |
| depression | F332 |
| depression | F331 |
| depression | F330 |
| depression | F33 |
| depression | F329 |
| depression | F328 |
| depression | F323 |
| depression | F322 |
| depression | F321 |
| depression | F320 |
| depression | F32 |
| diabetes | O243 |
| diabetes | O242 |
| diabetes | O241 |
| diabetes | O240 |
| diabetes | N083 |
| diabetes | M142 |
| diabetes | H360 |
| diabetes | H280 |
| diabetes | G632 |
| diabetes | G590 |
| diabetes | E14 |
| diabetes | E13 |
| diabetes | E12 |
| diabetes | E11 |
| diabetes | E10 |
| dyslipidaemia | E78 |
| eating disorder | F50.9 |
| eating disorder | F50.8 |
| eating disorder | F50.5 |
| eating disorder | F50.4 |
| eating disorder | F50.3 |
| eating disorder | F50.2 |
| eating disorder | F50.1 |
| eating disorder | F50.0 |
| epilepsy | G41 |
| epilepsy | G40 |
| gout | M140 |
| gout | M10 |
| heart failure | U31.9 |
| heart failure | U31.8 |
| heart failure | U31.1 |
| heart failure | U31 |
| heart failure | K62.4 |
| heart failure | K62.3 |
| heart failure | K62.2 |
| heart failure | K62.1 |
| heart failure | K62 |
| heart failure | K61.9 |
| heart failure | K61.8 |
| heart failure | K61.7 |
| heart failure | K61.6 |
| heart failure | K61.5 |
| heart failure | K61.4 |
| heart failure | K61.3 |
| heart failure | K61.2 |
| heart failure | K61.1 |
| heart failure | K61 |
| heart failure | K60.9 |
| heart failure | K60.8 |
| heart failure | K60.7 |
| heart failure | K60.6 |
| heart failure | K60.5 |
| heart failure | K60.4 |
| heart failure | K60.3 |
| heart failure | K60.2 |
| heart failure | K60.1 |
| heart failure | K60 |
| heart failure | K59.9 |
| heart failure | K59.8 |
| heart failure | K59.6 |
| heart failure | K59.5 |
| heart failure | K59.4 |
| heart failure | K59.3 |
| heart failure | K59.2 |
| heart failure | K59.1 |
| heart failure | K59 |
| heart failure | K24.6 |
| heart failure | K24.5 |
| heart failure | K24.1 |
| heart failure | I50.9 |
| heart failure | I50.1 |
| heart failure | I50 |
| heart failure | I43* |
| heart failure | I42.0 |
| heart failure | I42 |
| heart failure | I260 |
| heart failure | I132 |
| heart failure | I130 |
| heart failure | I110 |
| hiv/aids and sequelae | Z21 |
| hiv/aids and sequelae | R75 |
| hiv/aids and sequelae | O980 |
| hiv/aids and sequelae | N741 |
| hiv/aids and sequelae | N740 |
| hiv/aids and sequelae | N330 |
| hiv/aids and sequelae | M900 |
| hiv/aids and sequelae | M490 |
| hiv/aids and sequelae | M011 |
| hiv/aids and sequelae | K930 |
| hiv/aids and sequelae | K673 |
| hiv/aids and sequelae | K230 |
| hiv/aids and sequelae | J65 |
| hiv/aids and sequelae | F024 |
| hiv/aids and sequelae | C865 |
| hiv/aids and sequelae | C837 |
| hiv/aids and sequelae | C46 |
| hiv/aids and sequelae | B90 |
| hiv/aids and sequelae | B582 |
| hiv/aids and sequelae | B453 |
| hiv/aids and sequelae | B451 |
| hiv/aids and sequelae | B417 |
| hiv/aids and sequelae | B393 |
| hiv/aids and sequelae | B387 |
| hiv/aids and sequelae | B384 |
| hiv/aids and sequelae | B250 |
| hiv/aids and sequelae | B25 |
| hiv/aids and sequelae | B24 |
| hiv/aids and sequelae | B23 |
| hiv/aids and sequelae | B227 |
| hiv/aids and sequelae | B222 |
| hiv/aids and sequelae | B221 |
| hiv/aids and sequelae | B220 |
| hiv/aids and sequelae | B219 |
| hiv/aids and sequelae | B218 |
| hiv/aids and sequelae | B217 |
| hiv/aids and sequelae | B213 |
| hiv/aids and sequelae | B212 |
| hiv/aids and sequelae | B211 |
| hiv/aids and sequelae | B210 |
| hiv/aids and sequelae | B209 |
| hiv/aids and sequelae | B208 |
| hiv/aids and sequelae | B207 |
| hiv/aids and sequelae | B206 |
| hiv/aids and sequelae | B205 |
| hiv/aids and sequelae | B204 |
| hiv/aids and sequelae | B203 |
| hiv/aids and sequelae | B202 |
| hiv/aids and sequelae | B201 |
| hiv/aids and sequelae | B200 |
| hiv/aids and sequelae | A812 |
| hiv/aids and sequelae | A19 |
| hiv/aids and sequelae | A18 |
| hiv/aids and sequelae | A17 |
| hiv/aids and sequelae | A16 |
| hiv/aids and sequelae | A15 |
| hypertension | O10 |
| hypertension | I15 |
| hypertension | I13 |
| hypertension | I12 |
| hypertension | I11 |
| hypertension | I10 |
| ischaemic heart disease | X509 |
| ischaemic heart disease | X508 |
| ischaemic heart disease | X503 |
| ischaemic heart disease | X50 |
| ischaemic heart disease | L97.2 |
| ischaemic heart disease | K75.9 |
| ischaemic heart disease | K75.8 |
| ischaemic heart disease | K75.4 |
| ischaemic heart disease | K75.3 |
| ischaemic heart disease | K75.2 |
| ischaemic heart disease | K75.1 |
| ischaemic heart disease | K75 |
| ischaemic heart disease | K599 |
| ischaemic heart disease | K598 |
| ischaemic heart disease | K596 |
| ischaemic heart disease | K595 |
| ischaemic heart disease | K594 |
| ischaemic heart disease | K593 |
| ischaemic heart disease | K592 |
| ischaemic heart disease | K591 |
| ischaemic heart disease | K59 |
| ischaemic heart disease | K50.9 |
| ischaemic heart disease | K50.8 |
| ischaemic heart disease | K50.4 |
| ischaemic heart disease | K50.3 |
| ischaemic heart disease | K50.2 |
| ischaemic heart disease | K50.1 |
| ischaemic heart disease | K50 |
| ischaemic heart disease | K49.9 |
| ischaemic heart disease | K49.8 |
| ischaemic heart disease | K49.4 |
| ischaemic heart disease | K49.3 |
| ischaemic heart disease | K49.2 |
| ischaemic heart disease | K49.1 |
| ischaemic heart disease | K49 |
| ischaemic heart disease | K48.3 |
| ischaemic heart disease | K47.1 |
| ischaemic heart disease | K46.9 |
| ischaemic heart disease | K46.8 |
| ischaemic heart disease | K46.5 |
| ischaemic heart disease | K46.4 |
| ischaemic heart disease | K46.3 |
| ischaemic heart disease | K46.2 |
| ischaemic heart disease | K46.1 |
| ischaemic heart disease | K46 |
| ischaemic heart disease | K45.9 |
| ischaemic heart disease | K45.8 |
| ischaemic heart disease | K45.6 |
| ischaemic heart disease | K45.5 |
| ischaemic heart disease | K45.4 |
| ischaemic heart disease | K45.3 |
| ischaemic heart disease | K45.2 |
| ischaemic heart disease | K45.1 |
| ischaemic heart disease | K45 |
| ischaemic heart disease | K44.9 |
| ischaemic heart disease | K44.8 |
| ischaemic heart disease | K44.2 |
| ischaemic heart disease | K44.1 |
| ischaemic heart disease | K44 |
| ischaemic heart disease | K43.9 |
| ischaemic heart disease | K43.8 |
| ischaemic heart disease | K43.4 |
| ischaemic heart disease | K43.3 |
| ischaemic heart disease | K43.2 |
| ischaemic heart disease | K43.1 |
| ischaemic heart disease | K43 |
| ischaemic heart disease | K42.9 |
| ischaemic heart disease | K42.8 |
| ischaemic heart disease | K42.4 |
| ischaemic heart disease | K42.3 |
| ischaemic heart disease | K42.2 |
| ischaemic heart disease | K42.1 |
| ischaemic heart disease | K42 |
| ischaemic heart disease | K41.9 |
| ischaemic heart disease | K41.8 |
| ischaemic heart disease | K41.4 |
| ischaemic heart disease | K41.3 |
| ischaemic heart disease | K41.2 |
| ischaemic heart disease | K41.1 |
| ischaemic heart disease | K41 |
| ischaemic heart disease | K40.9 |
| ischaemic heart disease | K40.8 |
| ischaemic heart disease | K40.4 |
| ischaemic heart disease | K40.3 |
| ischaemic heart disease | K40.2 |
| ischaemic heart disease | K40.1 |
| ischaemic heart disease | K40 |
| ischaemic heart disease | I25 |
| ischaemic heart disease | I24.1 |
| ischaemic heart disease | I24 |
| ischaemic heart disease | I23 |
| ischaemic heart disease | I22 |
| ischaemic heart disease | I21 |
| ischaemic heart disease | I20 |
| learning disorder | F89 |
| learning disorder | F88 |
| learning disorder | F84.0 |
| learning disorder | F84 |
| learning disorder | F83 |
| learning disorder | F82 |
| learning disorder | F81 |
| learning disorder | F80 |
| learning disorder | F79 |
| learning disorder | F78 |
| learning disorder | F73 |
| learning disorder | F72 |
| learning disorder | F71 |
| learning disorder | F70 |
| liver disease | K77* |
| liver disease | K76.7 |
| liver disease | K76.6 |
| liver disease | K76 |
| liver disease | K75 |
| liver disease | K74.6 |
| liver disease | K74.5 |
| liver disease | K74.4 |
| liver disease | K74.3 |
| liver disease | K74.2 |
| liver disease | K74.0 |
| liver disease | K74 |
| liver disease | K73 |
| liver disease | K72.9 |
| liver disease | K72.1 |
| liver disease | K72 |
| liver disease | K71.7 |
| liver disease | K71 |
| liver disease | K70.3 |
| liver disease | K70.2 |
| liver disease | K70 |
| liver disease | I85 |
| metabolic disease (obesity) | E66 |
| neoplasm (blood) | D46.9 |
| neoplasm (blood) | D46.7 |
| neoplasm (blood) | D46.6 |
| neoplasm (blood) | D46.5 |
| neoplasm (blood) | D46.4 |
| neoplasm (blood) | D46.3 |
| neoplasm (blood) | D46.2 |
| neoplasm (blood) | D46.1 |
| neoplasm (blood) | D46.0 |
| neoplasm (blood) | D46 |
| neoplasm (blood) | D45 |
| neoplasm (blood) | C95 |
| neoplasm (blood) | C947 |
| neoplasm (blood) | C946 |
| neoplasm (blood) | C944 |
| neoplasm (blood) | C943 |
| neoplasm (blood) | C942 |
| neoplasm (blood) | C940 |
| neoplasm (blood) | C93 |
| neoplasm (blood) | C92 |
| neoplasm (blood) | C919 |
| neoplasm (blood) | C918 |
| neoplasm (blood) | C917 |
| neoplasm (blood) | C916 |
| neoplasm (blood) | C913 |
| neoplasm (blood) | C911 |
| neoplasm (blood) | C910 |
| neoplasm (blood) | C903 |
| neoplasm (blood) | C902 |
| neoplasm (blood) | C901 |
| neoplasm (blood) | C900 |
| neoplasm (blood) | C884 |
| neoplasm (blood) | C882 |
| neoplasm (blood) | C880 |
| neoplasm (blood) | C86 |
| neoplasm (ear, nose and throat) | D14.2 |
| neoplasm (ear, nose and throat) | D14.1 |
| neoplasm (ear, nose and throat) | D14.0 |
| neoplasm (ear, nose and throat) | D11.9 |
| neoplasm (ear, nose and throat) | D11.7 |
| neoplasm (ear, nose and throat) | D11.0 |
| neoplasm (ear, nose and throat) | D11 |
| neoplasm (ear, nose and throat) | D10.9 |
| neoplasm (ear, nose and throat) | D10.7 |
| neoplasm (ear, nose and throat) | D10.6 |
| neoplasm (ear, nose and throat) | D10.5 |
| neoplasm (ear, nose and throat) | D10.4 |
| neoplasm (ear, nose and throat) | D10.3 |
| neoplasm (ear, nose and throat) | D10.2 |
| neoplasm (ear, nose and throat) | D10.1 |
| neoplasm (ear, nose and throat) | D10 |
| neoplasm (ear, nose and throat) | D02.1 |
| neoplasm (ear, nose and throat) | D02.0 |
| neoplasm (ear, nose and throat) | D00.0 |
| neoplasm (ear, nose and throat) | C32 |
| neoplasm (ear, nose and throat) | C31 |
| neoplasm (ear, nose and throat) | C30 |
| neoplasm (ear, nose and throat) | C14 |
| neoplasm (ear, nose and throat) | C13 |
| neoplasm (ear, nose and throat) | C12 |
| neoplasm (ear, nose and throat) | C11 |
| neoplasm (ear, nose and throat) | C10 |
| neoplasm (ear, nose and throat) | C09 |
| neoplasm (ear, nose and throat) | C08 |
| neoplasm (ear, nose and throat) | C07 |
| neoplasm (ear, nose and throat) | C06 |
| neoplasm (ear, nose and throat) | C05 |
| neoplasm (ear, nose and throat) | C04 |
| neoplasm (ear, nose and throat) | C03 |
| neoplasm (ear, nose and throat) | C02 |
| neoplasm (ear, nose and throat) | C01 |
| neoplasm (liver) | D13.4 |
| neoplasm (liver) | D01.5 |
| neoplasm (liver) | C22 |
| neoplasm (lung) | D14.3 |
| neoplasm (lung) | D02.2 |
| neoplasm (lung) | C34 |
| neoplasm (lymphatics) | C96 |
| neoplasm (lymphatics) | C915 |
| neoplasm (lymphatics) | C914 |
| neoplasm (lymphatics) | C889 |
| neoplasm (lymphatics) | C887 |
| neoplasm (lymphatics) | C883 |
| neoplasm (lymphatics) | C85 |
| neoplasm (lymphatics) | C84 |
| neoplasm (lymphatics) | C83 |
| neoplasm (lymphatics) | C82 |
| neoplasm (lymphatics) | C81 |
| neoplasm (metastasis) | C79 |
| neoplasm (metastasis) | C78 |
| neoplasm (metastasis) | C77 |
| neoplasm (oesophagus) | D13.0 |
| neoplasm (oesophagus) | D00.1 |
| neoplasm (oesophagus) | C15 |
| neoplasm (ovary) | D27 |
| neoplasm (ovary) | C56 |
| neoplasm (pancreas) | D13.7 |
| neoplasm (pancreas) | D13.6 |
| neoplasm (pancreas) | C25 |
| neoplasm (pancreas) | D29.1 |
| neoplasm (pancreas) | D07.5 |
| neoplasm (pancreas) | C61 |
| neoplasm (rectum) | D12.8 |
| neoplasm (rectum) | D12.7 |
| neoplasm (rectum) | D01.2 |
| neoplasm (rectum) | D01.1 |
| neoplasm (rectum) | C20 |
| neoplasm (rectum) | C19 |
| neoplasm (rectum) | D30.1 |
| neoplasm (rectum) | D30.0 |
| neoplasm (rectum) | C65 |
| neoplasm (rectum) | C64 |
| neoplasm (skin) | D23.9 |
| neoplasm (skin) | D23.7 |
| neoplasm (skin) | D23.6 |
| neoplasm (skin) | D23.5 |
| neoplasm (skin) | D23.4 |
| neoplasm (skin) | D23.3 |
| neoplasm (skin) | D23.2 |
| neoplasm (skin) | D23.1 |
| neoplasm (skin) | D23.0 |
| neoplasm (skin) | D23 |
| neoplasm (skin) | D22.9 |
| neoplasm (skin) | D22.7 |
| neoplasm (skin) | D22.6 |
| neoplasm (skin) | D22.5 |
| neoplasm (skin) | D22.4 |
| neoplasm (skin) | D22.3 |
| neoplasm (skin) | D22.2 |
| neoplasm (skin) | D22.1 |
| neoplasm (skin) | D22.0 |
| neoplasm (skin) | D22 |
| neoplasm (skin) | D17.3 |
| neoplasm (skin) | D17.2 |
| neoplasm (skin) | D17.1 |
| neoplasm (skin) | D17.0 |
| neoplasm (skin) | D04.9 |
| neoplasm (skin) | D04.8 |
| neoplasm (skin) | D04.7 |
| neoplasm (skin) | D04.6 |
| neoplasm (skin) | D04.5 |
| neoplasm (skin) | D04.4 |
| neoplasm (skin) | D04.3 |
| neoplasm (skin) | D04.2 |
| neoplasm (skin) | D04.1 |
| neoplasm (skin) | D04.0 |
| neoplasm (skin) | D04 |
| neoplasm (skin) | D03.9 |
| neoplasm (skin) | D03.8 |
| neoplasm (skin) | D03.7 |
| neoplasm (skin) | D03.6 |
| neoplasm (skin) | D03.5 |
| neoplasm (skin) | D03.4 |
| neoplasm (skin) | D03.3 |
| neoplasm (skin) | D03.2 |
| neoplasm (skin) | D03.1 |
| neoplasm (skin) | D03.0 |
| neoplasm (skin) | D03 |
| neoplasm (skin) | C44 |
| neoplasm (stomach) | D13.1 |
| neoplasm (stomach) | D00.2 |
| neoplasm (stomach) | C16 |
| neoplasm (unspecified) | D17.9 |
| neoplasm (unspecified) | D09.9 |
| neoplasm (unspecified) | D01 |
| neoplastic disease (bladder) | D30.3 |
| neoplastic disease (bladder) | D09.0 |
| neoplastic disease (bladder) | C67 |
| neoplastic disease (breast) | D24 |
| neoplastic disease (breast) | D05.9 |
| neoplastic disease (breast) | D05.7 |
| neoplastic disease (breast) | D05.1 |
| neoplastic disease (breast) | D05.0 |
| neoplastic disease (breast) | D05 |
| neoplastic disease (breast) | C50 |
| neoplastic disease (cervix) | D26.9 |
| neoplastic disease (cervix) | D26.7 |
| neoplastic disease (cervix) | D26.1 |
| neoplastic disease (cervix) | D26.0 |
| neoplastic disease (cervix) | D06.9 |
| neoplastic disease (cervix) | D06.7 |
| neoplastic disease (cervix) | D06.1 |
| neoplastic disease (cervix) | D06.0 |
| neoplastic disease (cervix) | D06 |
| neoplastic disease (cervix) | C53 |
| neoplastic disease (colon) | D12.6 |
| neoplastic disease (colon) | D12.5 |
| neoplastic disease (colon) | D12.4 |
| neoplastic disease (colon) | D12.3 |
| neoplastic disease (colon) | D12.2 |
| neoplastic disease (colon) | D01.0 |
| neoplastic disease (colon) | C18 |
| other neoplasm | D48.9 |
| other neoplasm | D48.7 |
| other neoplasm | D48.6 |
| other neoplasm | D48.5 |
| other neoplasm | D48.4 |
| other neoplasm | D48.3 |
| other neoplasm | D48.2 |
| other neoplasm | D48.1 |
| other neoplasm | D48.0 |
| other neoplasm | D48 |
| other neoplasm | D47.9 |
| other neoplasm | D47.7 |
| other neoplasm | D47.5 |
| other neoplasm | D47.4 |
| other neoplasm | D47.3 |
| other neoplasm | D47.2 |
| other neoplasm | D47.1 |
| other neoplasm | D47.0 |
| other neoplasm | D47 |
| other neoplasm | D44.9 |
| other neoplasm | D44.8 |
| other neoplasm | D44.7 |
| other neoplasm | D44.6 |
| other neoplasm | D44.5 |
| other neoplasm | D44.4 |
| other neoplasm | D44.3 |
| other neoplasm | D44.2 |
| other neoplasm | D44.1 |
| other neoplasm | D44.0 |
| other neoplasm | D44 |
| other neoplasm | D43.9 |
| other neoplasm | D43.7 |
| other neoplasm | D43.4 |
| other neoplasm | D43.3 |
| other neoplasm | D43.2 |
| other neoplasm | D43.1 |
| other neoplasm | D43.0 |
| other neoplasm | D43 |
| other neoplasm | D42.9 |
| other neoplasm | D42.1 |
| other neoplasm | D42.0 |
| other neoplasm | D42 |
| other neoplasm | D41.9 |
| other neoplasm | D41.7 |
| other neoplasm | D41.4 |
| other neoplasm | D41.3 |
| other neoplasm | D41.2 |
| other neoplasm | D41.1 |
| other neoplasm | D41.0 |
| other neoplasm | D41 |
| other neoplasm | D40.9 |
| other neoplasm | D40.7 |
| other neoplasm | D40.1 |
| other neoplasm | D40.0 |
| other neoplasm | D40 |
| other neoplasm | D39.9 |
| other neoplasm | D39.7 |
| other neoplasm | D39.2 |
| other neoplasm | D39.1 |
| other neoplasm | D39.0 |
| other neoplasm | D39 |
| other neoplasm | D38.6 |
| other neoplasm | D38.5 |
| other neoplasm | D38.4 |
| other neoplasm | D38.3 |
| other neoplasm | D38.2 |
| other neoplasm | D38.1 |
| other neoplasm | D38.0 |
| other neoplasm | D38 |
| other neoplasm | D37.9 |
| other neoplasm | D37.7 |
| other neoplasm | D37.6 |
| other neoplasm | D37.5 |
| other neoplasm | D37.4 |
| other neoplasm | D37.3 |
| other neoplasm | D37.2 |
| other neoplasm | D37.1 |
| other neoplasm | D37.0 |
| other neoplasm | D37 |
| other neoplasm | D36.9 |
| other neoplasm | D36.7 |
| other neoplasm | D36.1 |
| other neoplasm | D36.0 |
| other neoplasm | D36 |
| other neoplasm | D35.9 |
| other neoplasm | D35.8 |
| other neoplasm | D35.7 |
| other neoplasm | D35.6 |
| other neoplasm | D35.5 |
| other neoplasm | D35.4 |
| other neoplasm | D35.3 |
| other neoplasm | D35.2 |
| other neoplasm | D35.1 |
| other neoplasm | D35.0 |
| other neoplasm | D35 |
| other neoplasm | D34 |
| other neoplasm | D33.9 |
| other neoplasm | D33.7 |
| other neoplasm | D33.4 |
| other neoplasm | D33.3 |
| other neoplasm | D33.2 |
| other neoplasm | D33.1 |
| other neoplasm | D33.0 |
| other neoplasm | D33 |
| other neoplasm | D32.9 |
| other neoplasm | D32.1 |
| other neoplasm | D32.0 |
| other neoplasm | D32 |
| other neoplasm | D31.9 |
| other neoplasm | D31.6 |
| other neoplasm | D31.5 |
| other neoplasm | D31.4 |
| other neoplasm | D31.3 |
| other neoplasm | D31.2 |
| other neoplasm | D31.1 |
| other neoplasm | D31.0 |
| other neoplasm | D31 |
| other neoplasm | D21.9 |
| other neoplasm | D21.6 |
| other neoplasm | D21.5 |
| other neoplasm | D21.4 |
| other neoplasm | D21.3 |
| other neoplasm | D21.2 |
| other neoplasm | D21.1 |
| other neoplasm | D21.0 |
| other neoplasm | D21 |
| other neoplasm | D19.9 |
| other neoplasm | D19.7 |
| other neoplasm | D19 |
| other neoplasm | D18.1 |
| other neoplasm | D18.0 |
| other neoplasm | D18 |
| other neoplasm | D17.7 |
| other neoplasm | D17.5 |
| other neoplasm | D17.4 |
| other neoplasm | D17 |
| other neoplasm | D16.9 |
| other neoplasm | D16.8 |
| other neoplasm | D16.7 |
| other neoplasm | D16.6 |
| other neoplasm | D16.5 |
| other neoplasm | D16.4 |
| other neoplasm | D16.3 |
| other neoplasm | D16.2 |
| other neoplasm | D16.1 |
| other neoplasm | D16.0 |
| other neoplasm | D16 |
| other neoplasm | D15.9 |
| other neoplasm | D15.7 |
| other neoplasm | D15.2 |
| other neoplasm | D15.1 |
| other neoplasm | D15.0 |
| other neoplasm | D15 |
| other neoplasm | D10.0 |
| other neoplasm | D09.7 |
| other neoplasm | D09.3 |
| other neoplasm | D09.2 |
| other neoplasm | D09 |
| other neoplasm | D07 |
| other neoplasm | C97 |
| other neoplasm | C80 |
| other neoplasm | C76 |
| other neoplasm | C75 |
| other neoplasm | C74 |
| other neoplasm | C73 |
| other neoplasm | C72 |
| other neoplasm | C71 |
| other neoplasm | C70 |
| other neoplasm | C69 |
| other neoplasm | C49 |
| other neoplasm | C48 |
| other neoplasm | C47 |
| other neoplasm | C46 |
| other neoplasm | C45 |
| other neoplasm | C43 |
| other neoplasm | C41 |
| other neoplasm | C40 |
| other neoplasm | C39 |
| other neoplasm | C38 |
| other neoplasm | C37 |
| other neoplasm | C00 |
| other neoplasm (female reproductive organ) | D28.9 |
| other neoplasm (female reproductive organ) | D28.7 |
| other neoplasm (female reproductive organ) | D28.2 |
| other neoplasm (female reproductive organ) | D28.1 |
| other neoplasm (female reproductive organ) | D28.0 |
| other neoplasm (female reproductive organ) | D28 |
| other neoplasm (female reproductive organ) | D26 |
| other neoplasm (female reproductive organ) | D25.9 |
| other neoplasm (female reproductive organ) | D25.2 |
| other neoplasm (female reproductive organ) | D25.1 |
| other neoplasm (female reproductive organ) | D25.0 |
| other neoplasm (female reproductive organ) | D25 |
| other neoplasm (female reproductive organ) | D07.3 |
| other neoplasm (female reproductive organ) | D07.2 |
| other neoplasm (female reproductive organ) | D07.1 |
| other neoplasm (female reproductive organ) | D07.0 |
| other neoplasm (female reproductive organ) | C58 |
| other neoplasm (female reproductive organ) | C57 |
| other neoplasm (female reproductive organ) | C55 |
| other neoplasm (female reproductive organ) | C54 |
| other neoplasm (female reproductive organ) | C52 |
| other neoplasm (female reproductive organ) | C51 |
| other neoplasm (gastrointestinal tract) | D20.1 |
| other neoplasm (gastrointestinal tract) | D20.0 |
| other neoplasm (gastrointestinal tract) | D20 |
| other neoplasm (gastrointestinal tract) | D19.1 |
| other neoplasm (gastrointestinal tract) | D13.9 |
| other neoplasm (gastrointestinal tract) | D13.5 |
| other neoplasm (gastrointestinal tract) | D13.3 |
| other neoplasm (gastrointestinal tract) | D13.2 |
| other neoplasm (gastrointestinal tract) | D13 |
| other neoplasm (gastrointestinal tract) | D12.9 |
| other neoplasm (gastrointestinal tract) | D12.1 |
| other neoplasm (gastrointestinal tract) | D12.0 |
| other neoplasm (gastrointestinal tract) | D12 |
| other neoplasm (gastrointestinal tract) | D01.9 |
| other neoplasm (gastrointestinal tract) | D01.7 |
| other neoplasm (gastrointestinal tract) | D01.4 |
| other neoplasm (gastrointestinal tract) | D01.3 |
| other neoplasm (gastrointestinal tract) | C26 |
| other neoplasm (gastrointestinal tract) | C24 |
| other neoplasm (gastrointestinal tract) | C23 |
| other neoplasm (gastrointestinal tract) | C21 |
| other neoplasm (gastrointestinal tract) | C17 |
| other neoplasm (male reproductive organ) | D29.9 |
| other neoplasm (male reproductive organ) | D29.7 |
| other neoplasm (male reproductive organ) | D29.4 |
| other neoplasm (male reproductive organ) | D29.3 |
| other neoplasm (male reproductive organ) | D29.2 |
| other neoplasm (male reproductive organ) | D29.0 |
| other neoplasm (male reproductive organ) | D29 |
| other neoplasm (male reproductive organ) | D17.6 |
| other neoplasm (male reproductive organ) | D07.6 |
| other neoplasm (male reproductive organ) | D07.4 |
| other neoplasm (male reproductive organ) | C63 |
| other neoplasm (male reproductive organ) | C62 |
| other neoplasm (male reproductive organ) | C60 |
| other neoplasm (respiratory tract) | D19.0 |
| other neoplasm (respiratory tract) | D14.4 |
| other neoplasm (respiratory tract) | D14 |
| other neoplasm (respiratory tract) | D02.4 |
| other neoplasm (respiratory tract) | D02.3 |
| other neoplasm (respiratory tract) | D02 |
| other neoplasm (respiratory tract) | C33 |
| other neoplasm (urinary tract) | D30.9 |
| other neoplasm (urinary tract) | D30.7 |
| other neoplasm (urinary tract) | D30.4 |
| other neoplasm (urinary tract) | D30.2 |
| other neoplasm (urinary tract) | D30 |
| other neoplasm (urinary tract) | D09.1 |
| other neoplasm (urinary tract) | C68 |
| other neoplasm (urinary tract) | C66 |
| paraplegia | G82.2 |
| paraplegia | G82.1 |
| paraplegia | G82.0 |
| peptic ulcer disease | K28 |
| peptic ulcer disease | K27 |
| peptic ulcer disease | K26 |
| peptic ulcer disease | K25 |
| peripheral arterial disease | L65.3 |
| peripheral arterial disease | L65.2 |
| peripheral arterial disease | L65.1 |
| peripheral arterial disease | L65 |
| peripheral arterial disease | L63.9 |
| peripheral arterial disease | L63.8 |
| peripheral arterial disease | L63.5 |
| peripheral arterial disease | L63.3 |
| peripheral arterial disease | L63.2 |
| peripheral arterial disease | L63.1 |
| peripheral arterial disease | L63 |
| peripheral arterial disease | L62.9 |
| peripheral arterial disease | L62.8 |
| peripheral arterial disease | L62.2 |
| peripheral arterial disease | L62.1 |
| peripheral arterial disease | L62 |
| peripheral arterial disease | L60.9 |
| peripheral arterial disease | L60.8 |
| peripheral arterial disease | L60.4 |
| peripheral arterial disease | L60.3 |
| peripheral arterial disease | L60.2 |
| peripheral arterial disease | L60.1 |
| peripheral arterial disease | L60 |
| peripheral arterial disease | L59.9 |
| peripheral arterial disease | L59.8 |
| peripheral arterial disease | L59.7 |
| peripheral arterial disease | L59.6 |
| peripheral arterial disease | L59.5 |
| peripheral arterial disease | L59.4 |
| peripheral arterial disease | L59.3 |
| peripheral arterial disease | L59.2 |
| peripheral arterial disease | L59.1 |
| peripheral arterial disease | L59 |
| peripheral arterial disease | L58.9 |
| peripheral arterial disease | L58.8 |
| peripheral arterial disease | L58.7 |
| peripheral arterial disease | L58.6 |
| peripheral arterial disease | L58.5 |
| peripheral arterial disease | L58.4 |
| peripheral arterial disease | L58.3 |
| peripheral arterial disease | L58.2 |
| peripheral arterial disease | L58.1 |
| peripheral arterial disease | L58 |
| peripheral arterial disease | L54.9 |
| peripheral arterial disease | L54.8 |
| peripheral arterial disease | L54.4 |
| peripheral arterial disease | L54.2 |
| peripheral arterial disease | L54.1 |
| peripheral arterial disease | L54 |
| peripheral arterial disease | L53.9 |
| peripheral arterial disease | L53.8 |
| peripheral arterial disease | L53.2 |
| peripheral arterial disease | L53.1 |
| peripheral arterial disease | L53 |
| peripheral arterial disease | L52.9 |
| peripheral arterial disease | L52.8 |
| peripheral arterial disease | L52.2 |
| peripheral arterial disease | L52.1 |
| peripheral arterial disease | L52 |
| peripheral arterial disease | L51.9 |
| peripheral arterial disease | L51.8 |
| peripheral arterial disease | L51.6 |
| peripheral arterial disease | L51.5 |
| peripheral arterial disease | L51.4 |
| peripheral arterial disease | L51.3 |
| peripheral arterial disease | L51.2 |
| peripheral arterial disease | L51.1 |
| peripheral arterial disease | L51 |
| peripheral arterial disease | L50.9 |
| peripheral arterial disease | L50.8 |
| peripheral arterial disease | L50.6 |
| peripheral arterial disease | L50.5 |
| peripheral arterial disease | L50.4 |
| peripheral arterial disease | L50.3 |
| peripheral arterial disease | L50.2 |
| peripheral arterial disease | L50.1 |
| peripheral arterial disease | L50 |
| peripheral arterial disease | L289 |
| peripheral arterial disease | L288 |
| peripheral arterial disease | L286 |
| peripheral arterial disease | L285 |
| peripheral arterial disease | L282 |
| peripheral arterial disease | L281 |
| peripheral arterial disease | L28 |
| peripheral arterial disease | L279 |
| peripheral arterial disease | L278 |
| peripheral arterial disease | L276 |
| peripheral arterial disease | L275 |
| peripheral arterial disease | L272 |
| peripheral arterial disease | L271 |
| peripheral arterial disease | L27 |
| peripheral arterial disease | L269 |
| peripheral arterial disease | L268 |
| peripheral arterial disease | L267 |
| peripheral arterial disease | L266 |
| peripheral arterial disease | L265 |
| peripheral arterial disease | L263 |
| peripheral arterial disease | L262 |
| peripheral arterial disease | L261 |
| peripheral arterial disease | L26 |
| peripheral arterial disease | L259 |
| peripheral arterial disease | L258 |
| peripheral arterial disease | L254 |
| peripheral arterial disease | L252 |
| peripheral arterial disease | L251 |
| peripheral arterial disease | L25 |
| peripheral arterial disease | L236 |
| peripheral arterial disease | L235 |
| peripheral arterial disease | L233 |
| peripheral arterial disease | L232 |
| peripheral arterial disease | L231 |
| peripheral arterial disease | L23 |
| peripheral arterial disease | L224 |
| peripheral arterial disease | L223 |
| peripheral arterial disease | L222 |
| peripheral arterial disease | L219 |
| peripheral arterial disease | L218 |
| peripheral arterial disease | L215 |
| peripheral arterial disease | L214 |
| peripheral arterial disease | L213 |
| peripheral arterial disease | L21 |
| peripheral arterial disease | L209 |
| peripheral arterial disease | L208 |
| peripheral arterial disease | L206 |
| peripheral arterial disease | L205 |
| peripheral arterial disease | L204 |
| peripheral arterial disease | L203 |
| peripheral arterial disease | L20 |
| peripheral arterial disease | L199 |
| peripheral arterial disease | L198 |
| peripheral arterial disease | L196 |
| peripheral arterial disease | L195 |
| peripheral arterial disease | L194 |
| peripheral arterial disease | L193 |
| peripheral arterial disease | L19 |
| peripheral arterial disease | L189 |
| peripheral arterial disease | L188 |
| peripheral arterial disease | L186 |
| peripheral arterial disease | L185 |
| peripheral arterial disease | L184 |
| peripheral arterial disease | L183 |
| peripheral arterial disease | L18 |
| peripheral arterial disease | L169 |
| peripheral arterial disease | L168 |
| peripheral arterial disease | L16 |
| peripheral arterial disease | I79 |
| peripheral arterial disease | I78 |
| peripheral arterial disease | I77 |
| peripheral arterial disease | I745 |
| peripheral arterial disease | I744 |
| peripheral arterial disease | I743 |
| peripheral arterial disease | I74 |
| peripheral arterial disease | I739 |
| peripheral arterial disease | I738 |
| peripheral arterial disease | I731 |
| peripheral arterial disease | I730 |
| peripheral arterial disease | I73 |
| peripheral arterial disease | I72 |
| peripheral arterial disease | I72 |
| peripheral arterial disease | I72 |
| peripheral arterial disease | I719 |
| peripheral arterial disease | I719 |
| peripheral arterial disease | I718 |
| peripheral arterial disease | I718 |
| peripheral arterial disease | I716 |
| peripheral arterial disease | I716 |
| peripheral arterial disease | I715 |
| peripheral arterial disease | I715 |
| peripheral arterial disease | I714 |
| peripheral arterial disease | I714 |
| peripheral arterial disease | I713 |
| peripheral arterial disease | I713 |
| peripheral arterial disease | I712 |
| peripheral arterial disease | I712 |
| peripheral arterial disease | I711 |
| peripheral arterial disease | I711 |
| peripheral arterial disease | I710 |
| peripheral arterial disease | I710 |
| peripheral arterial disease | I71 |
| peripheral arterial disease | I70 |
| peripheral arterial disease | I70 |
| psychoses | F30.2 |
| psychoses | F29 |
| psychoses | F28 |
| psychoses | F23 |
| psychoses | F22 |
| psychoses | F09 |
| rheumatoid arthritis | M08.4 |
| rheumatoid arthritis | M08.3 |
| rheumatoid arthritis | M08.2 |
| rheumatoid arthritis | M08.0 |
| rheumatoid arthritis | M06.9 |
| rheumatoid arthritis | M06.8 |
| rheumatoid arthritis | M06.3 |
| rheumatoid arthritis | M06.2 |
| rheumatoid arthritis | M06.0 |
| rheumatoid arthritis | M05.9 |
| rheumatoid arthritis | M05.8 |
| rheumatoid arthritis | M05.3 |
| rheumatoid arthritis | M05.2 |
| rheumatoid arthritis | M05.1 |
| rheumatoid arthritis | M05.0 |
| rheumatoid arthritis | J99.0 |
| rheumatoid arthritis | I52.8 |
| schizophrenia | F29 |
| schizophrenia | F28 |
| schizophrenia | F25 |
| schizophrenia | F24 |
| schizophrenia | F23 |
| schizophrenia | F22 |
| schizophrenia | F21 |
| schizophrenia | F20 |
| stroke | U54.3 |
| stroke | L37.2 |
| stroke | L35.3 |
| stroke | L314 |
| stroke | L311 |
| stroke | L31.9 |
| stroke | L31.8 |
| stroke | L31.4 |
| stroke | L31.3 |
| stroke | L31.1 |
| stroke | L31 |
| stroke | L30.9 |
| stroke | L30.8 |
| stroke | L30.3 |
| stroke | L30.1 |
| stroke | L30 |
| stroke | L295 |
| stroke | L294 |
| stroke | L29.9 |
| stroke | L29.8 |
| stroke | L29.7 |
| stroke | L29.6 |
| stroke | L29.5 |
| stroke | L29.4 |
| stroke | L29.3 |
| stroke | L29.2 |
| stroke | L29.1 |
| stroke | L29 |
| stroke | I698 |
| stroke | I694 |
| stroke | I693 |
| stroke | I692 |
| stroke | I691 |
| stroke | I690 |
| stroke | I69 |
| stroke | I68 |
| stroke | I679 |
| stroke | I672 |
| stroke | I67 |
| stroke | I66 |
| stroke | I65 |
| stroke | I64 |
| stroke | I63 |
| stroke | I63 |
| stroke | I629 |
| stroke | I621 |
| stroke | I620 |
| stroke | I62 |
| stroke | I61 |
| stroke | I61 |
| stroke | I61 |
| stroke | I60 |
| stroke | I60 |
| stroke | I60 |
| stroke | G467 |
| stroke | G466 |
| stroke | G465 |
| stroke | G464 |
| stroke | G463 |
| substance abuse | F19 |
| substance abuse | F18 |
| substance abuse | F16 |
| substance abuse | F15 |
| substance abuse | F14 |
| substance abuse | F13 |
| substance abuse | F12 |
| substance abuse | F11 |
| substance abuse | F10 |
